# Global incidence and death estimates of chronic kidney disease due to hypertension from 1990 to 2019, an ecological analysis of the global burden of diseases 2019 study

**DOI:** 10.1186/s12882-023-03391-z

**Published:** 2023-11-29

**Authors:** Yan Liu, Qin He, Qiying Li, Min Tian, Xiaojiao Li, Xufeng Yao, Dongmei He, Chunying Deng

**Affiliations:** 1 Department of Nephrology, Chengdu Second People’s Hospital, No. 2 Huatai Road, Chenghua District, 610000 Chengdu, Sichuan Province China; 2 Department of Endocrine, The fourth people’s hospital of Zi Gong, No. 400, North Dangui Street, Ziliujing District, 643000 Zigong, Sichuan Province China; 3 Department of Stomatology, Traditional Chinese Medicine Hospital, No. 800 Zhongshan Street, 610000 Lishui City, Zhejiang Province China

**Keywords:** Chronic Kidney Disease due to Hypertension, Global burden, Age-standardized incidence rate, Age-standardized deaths rate, Average annual percentage change, Spatial autocorrelation analysis

## Abstract

**Background:**

The intricate relationship between hypertension and chronic kidney disease (CKD) presents a global challenge for prevention of hypertension-related CKD. This study’s objective is to analyze age, gender, regional disparities, and evolving trends in the disease burden of hypertension-related CKD. We aim to estimate changing spatial and temporal trends in incidence and mortality rates, considering the socio-demographic index (SDI), to inform health strategies effectively.

**Method:**

Age-standardized incidence rates (ASIR) and death rates (ASDR) were collected from the GBD 2019. Trend analysis was conducted by Joinpoint regression of ASRs from 1990 to 2019. Spatial autocorrelation analysis was performed to obtain spatial patterns. The association between SDI and burden of CKD due to hypertension was estimated using a Pearson correlation analysis.

**Results:**

The global ASIR and ASDR due to hypertension-related CKD were 19.45 (95% CI, 17.85 to 21.09) and 5.88 (95% CI, 4.95 to 6.82) per 100 K population in 2019, representing increases of 17.89% and 13.29% compared to 1990, respectively. The elderly population and males were found the highest ASIR and ASDR. The high SDI region had the highest ASIRs, while low SDI regions experienced the highest ASDRs. Joinpoint regression found both global ASIR and ASDR showed increasing trends, with the highest increases observed in middle- and high-SDI regions, respectively. The SDI exhibited a positive association with ASIRs but displayed an inverse V-shaped correlation with the average annual percentage change (AAPC) of ASIRs. Spatial autocorrelation analysis revel significant positive spatial autocorrelation for the AAPC of ASDRs and ASIRs, from 1990 to 2019.

**Conclusions:**

Results met the objectives, and demonstrated a rising global burden of hypertension-related CKD. Factors such as aging, gender, and regional variations should be considered when designing control measures and developing healthcare systems to effectively address the burden of this complex condition.

**Supplementary Information:**

The online version contains supplementary material available at 10.1186/s12882-023-03391-z.

## Background

Chronic kidney disease (CKD) is a prevalent non-communicable disease afflicting excess of 650 million people and resulting more than 1.2 million deaths in 2017 worldwide [1]. It has been well documented that the prevalence number of chronic kidney disease varies considerably across the world, such as, almost a third of patients living in China (132.3 million) and India (115.1 million) [1]. A significant contributor to the CKD burden is hypertension, recognized as a leading risk factor for the development and progression of kidney disease, which was reported affect up to 90% of the population with CKD and accelerating its progression [2, 3]. The intricate interplay between hypertension and kidney function has far-reaching implications for global health [4].

Hypertension, commonly known as high blood pressure, is a pervasive and modifiable risk factor, and its association with CKD has been well-established, especially in Low- and Middle-income countries [5, 6]. Uncontrolled hypertension can lead to kidney damage, ultimately culminating in CKD, which, in its advanced stages, necessitates life-long dialysis or kidney transplantation [7]. However, there is no consensus on the goal of optimal blood pressure (BP) in the relevant guidelines, which makes it difficult to prevent hypertension related CKD [8]. In addition, the uneven worldwide distribution of chronic diseases, such as CKD, complicates the allocation of global health resources [9, 10]. Global burden of disease (GBD) CKD Collaboration estimated the burden of CKD globally in 2017, and highlighted its rising incidence and deaths burden [1]. CKD with different etiologies (such as diabetes, glomerulonephritis, and hypertension, etc.) were not reported in depth, nor was the temporal trend over the past 30 years. Exploring the burden pattern of aetiology-specific CKD, and the temporal trends may promote the precise prevention of CKD via more targeted prevention strategies.

The global implications of hypertension-related kidney disease extend beyond clinical concerns, impacting healthcare systems, economies, and, most importantly, the quality of life of affected individuals. Thus, a comprehensive comparison for temporal and spatial patterns of hypertension-related kidney disease has become imperative. To achieve this, the Global Burden of Disease (GBD) framework has emerged as a robust tool for assessing the impact of diseases on a global scale, which evaluated 369 diseases and injuries in 204 countries and territories from 1990 to 2019 to illustrate the landscape of CKD caused by distinct etiologies [11].

In our study, we hypothesize that there are significant regional, age, and gender disparities in the incidence and mortality burden of global hypertensive kidney disease. This poses a significant challenge to the prevention and control of the global burden of chronic kidney disease. Therefore, our study will investigate the age and gender distribution of the incidence and death rates of hypertension-related CKD. And we will further explore the temporal trends of age standardized incidence and death rates of CKD due to hypertension, by worldwide, regional and countries. In addition, we will analyze the relationship between SDI and CKD due to hypertension burden, and finally explored the spatial characteristics in the CKD due to hypertension burden. We hope our findings can facilitate evidence-based decision-making for clinical practice, public health policy, and global health strategies to mitigate the global burden of hypertension-related kidney disease.

## Materials and methods

### Data source

Resources included in this study were collected from the official website of the GBD 2019 Study (http://ghdx.healthdata.org). The GBD 2019 study annually provides age-sex-specific incidence, prevalence, deaths, years of life lost (YLL), years lived with disability (YLD), and disability-adjusted life-years (DALYs) due to 369 diseases and injuries for 204 countries throughout the world. Detailed resources search, inclusion and exclusion criteria, data processing steps and modeling methods of GBD 2019 have been delineated in previous GBD study [11]. GBD complies with the Guidelines for Accurate and Transparent Health Estimates Reporting statement [12]. The data sources were estimated from mathematical models based on surveillance data rather than surveillance data itself, which allow researchers download in accordance with their study periods for secondary analysis, thereby obviating the need for ethics approval.

In our study, we downloaded the age specific and age standardized annual incidence and deaths rates, with 95% confidence intervals (CIs) based on age and sex from 1990 to 2019 of the CKD due to hypertension, which corresponded ICD9/10 codes according to the GBD Chronic Kidney Disease Collaboration [1], using the Global Health Data Exchange (GHDx) query tool (http://ghdx.healthdata.org/gbd-results-tool). The socio-demographic index (SDI) was also extracted from the Institute for Health Metrics and Evaluation (IHME) [13]. SDI was estimated based on the country development, and composite of the country’s total fertility rate for women younger than 25 years, educational attainment, and lag-distributed income per capita [14]. According to the corresponding SDI, 204 countries and territories were divided into five regions including low, low-middle, middle, high-middle, and high-SDI regions. Besides, the world was geographically categorized into 21 GBD areas, including Oceania, Central Asia, Central Europe, Australasia [15].

### Statistical analysis

We employed a Joinpoint regression analysis (National Cancer Institute Joinpoint regression program software, version 4.1.0) to investigate the temporal trends of ASIR and ASDR related to CKD attributed to hypertension from 1990 to 2019. To ascertain the significance of one or more Joinpoints, the Monte Carlo permutation method was employed. The AAPC and its corresponding 95% confidence interval (CI) were computed as a weighted average of the segmented trends [16]. Furthermore, to assess the correlation between ASR/AAPCs and the SDI at both regional and country levels using a Pearson correlation analysis. Relevant analysis and data visualizations were performed using R 4.0.3 software (Lucent Technologies, Jasmine Mountain, USA).

Furthermore, we conducted a spatial autocorrelation analysis to examine the spatial patterns of ASIR, ASDR, and AAPC across 204 countries, using arcgis10.8 (Environmental Systems Research Institute, US). To evaluate global spatial autocorrelation, we employed the global Moran’s I statistic, which ranges from − 1 to + 1 [17]. A positive Moran’s I value suggests that countries with high ASRs/AAPCs tend to be located near other countries with high ASRs/AAPCs, or countries with low ASRs/AAPCs are clustered together, while a negative Moran’s I value implies that countries with high ASRs/AAPCs tend to be near countries with low ASRs/AAPCs, or vice versa. Significance in spatial autocorrelation was determined by Z-scores greater than or equal to 1.96 and p-values less than 0.05 for Moran’s I [18]. In addition, local Moran’s index, known as the local index of spatial autocorrelation (LISA), was used to identify the existence of spatial clusters of countries with high or low values [17]. There can be five different scenarios within the LISA: (1) high-high, “hot spots”; (2) low-low, “cold spots”; (3) high-low, “spatial outliers”; (4) low-high, again “spatial outliers”; and (5) no significant local spatial autocorrelation. This methodology allows the identification of regions that contribute to spatial autocorrelation.

## Results

### Point estimation of chronic kidney disease burden in 2019

**Fig. 1:**
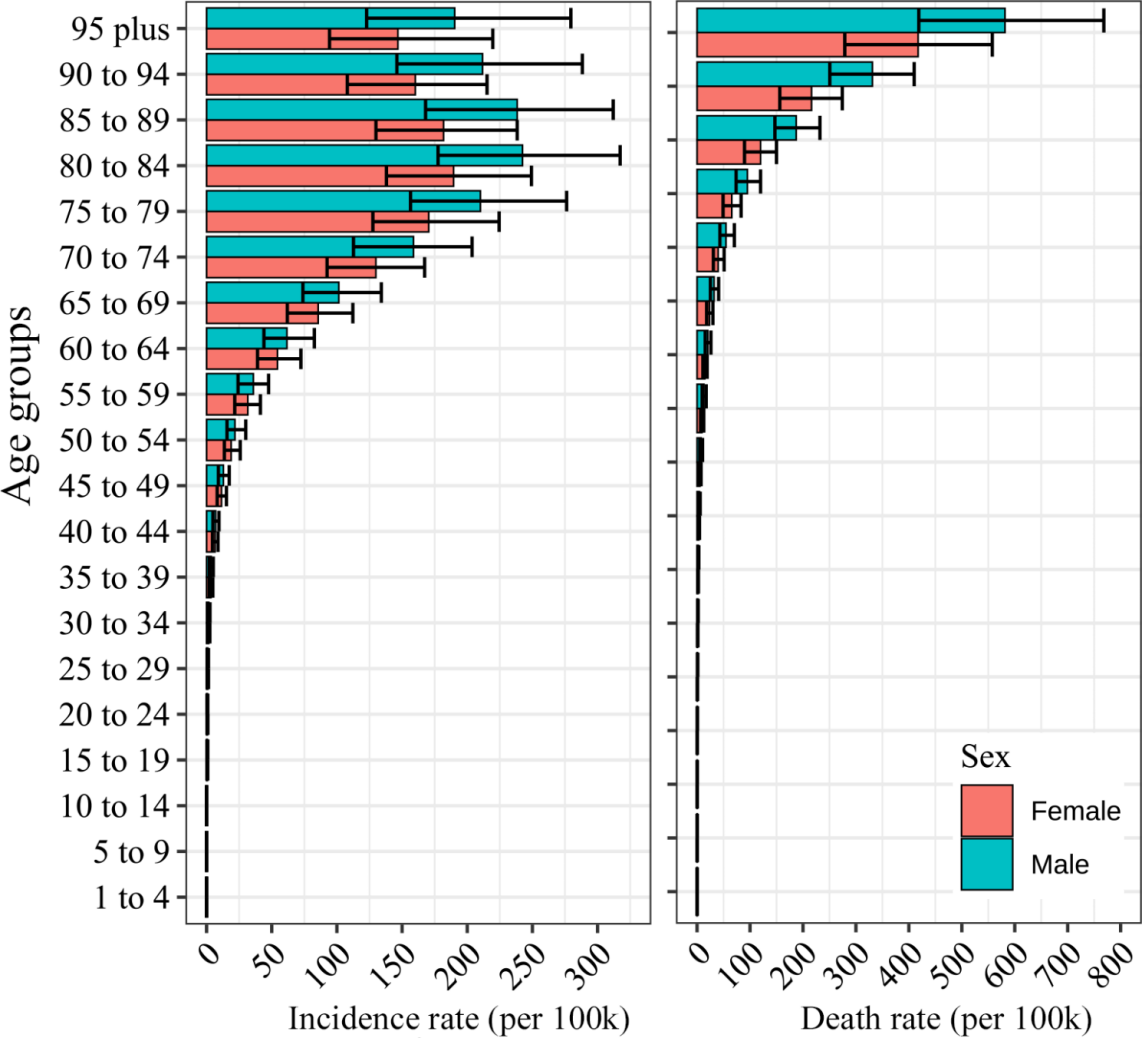
The age-specific incidence (**A**) and deaths (**B**) rates of CKD due to hypertension in different age groups, globally in 2019

Globally, the ASIR was 19.45 (95% CI, 17.85 to 21.09) per 100k population for both sexes, while ASDR was 5.88 (95% CI, 4.95 to 6.82). The ASIR of global female population was 17.84 (95% CI, 16.42 to 19.30) per 100k population, which was slightly lower than males (ASIR, 21.43; 95% CI, 19.67 to 23.28) (Table 1). Similar sex variations were observed in males with a higher ASDR than it in males (Table 1). In terms of age distribution, the highest incidence and deaths rates were all observed in the old population, and higher in males than females of all the age groups (Fig. 1). Similar results were observed in the five SDI regions (Supplementary Figs. 1–2).

**Table 1:** The ASIR, ASDR, and trends of CKD due to hypertension from 1990 to 2019, by global and regions

Location	Incidence			Death			AAPC of ASIR (1990 to 2019, %)	AAPC of ASDR (1990 to 2019, %)
	ASIR 2019 (Per 100 000 population)	ASIR 2019 (Per 100 000 population)	Percentage Change (%)#	ASDR 2019 (Per 100 K)	ASDR 2019 (Per 100 K)	Percentage Change (%)#		
**Global**								
Both	15.97 (14.6, 17.4)	19.45 (17.85, 21.09)	17.89	5.1 (4.35, 5.92)	5.88 (4.95, 6.82)	13.29	0.69 (0.66, 0.71)*	0.49 (0.42, 0.56)*
Female	15.00 (13.74, 17.38)	17.84 (16.42, 19.30)	15.88	4.25 (3.58, 5.00)	4.98 (4.11, 5.87)	14.51	0.6 (0.59, 0.61)*	0.54 (0.46, 0.62)*
Male	17.26 (15.79, 18.83)	21.43 (19.67, 23.28)	19.46	6.45 (5.43, 7.59)	7.06 (6.06, 8.25)	9.90	0.75 (0.74, 0.76)*	0.36 (0.2, 0.51)*
**SDI Regions**								
High SDI	22.08 (20.29, 24.01)	24.96 (23.02, 27.05)	11.56	3.37 (2.81, 3.92)	4.48 (3.63, 5.27)	24.94	0.42 (0.41, 0.44)*	0.99 (0.76, 1.21)*
High-middle SDI	13.65 (12.43, 14.94)	17.5 (15.97, 19.03)	21.96	3.73 (3.16, 4.37)	3.93 (3.27, 4.57)	5.06	0.86 (0.84, 0.88)*	0.17 (0.06, 0.28)*
Middle SDI	13.56 (12.3, 14.95)	18.85 (17.26, 20.52)	28.04	6.97 (5.91, 8.19)	7.58 (6.33, 8.79)	7.97	1.15 (1.13, 1.16)*	0.31 (0.21, 0.4)*
Low-middle SDI	11.69 (10.62, 12.85)	15.69 (14.28, 17.17)	25.47	6.49 (5.41, 7.84)	6.6 (5.46, 7.8)	1.64	1.01 (0.96, 1.06)*	0.06 (-0.09, 0.2)
Low SDI	9.73 (8.9, 10.62)	12.82 (11.64, 14.03)	24.14	9.25 (7.65, 11.22)	8.16 (6.85, 9.66)	-13.30	0.96 (0.93, 0.99)*	-0.42 (-0.52, -0.32)*
**Geographical Regions**								
East Asia	12.19 (10.94, 13.56)	14.11 (12.82, 15.54)	13.63	4.41 (3.68, 5.21)	4.22 (3.45, 5.02)	-4.65	0.51 (0.48, 0.54)*	-0.14 (-0.27, -0.01)*
South Asia	11.64 (10.53, 12.8)	14.59 (13.18, 16.01)	20.24	5.85 (4.51, 7.43)	5.34 (4.23, 6.58)	-9.62	0.79 (0.74, 0.85)*	-0.35 (-0.98, 0.28)
Southeast Asia	11.51 (10.49, 12.65)	17.5 (15.98, 19.09)	34.20	10.61(9.04, 12.5)	11.07 (9.29, 13.06)	4.18	1.45 (1.4, 1.49)*	0.17 (0, 0.34)*
Central Asia	8.15 (7.26, 9.14)	13.38 (11.99, 14.99)	39.09	1.01 (0.76, 1.36)	1.69 (1.26, 2.27)	40.42	1.74 (1.67, 1.8)*	1.84 (1.49, 2.19)*
High-income Asia Pacific	23.25 (21.38, 25.29)	25.23 (23.13, 27.42)	7.85	4.19 (3.37, 5)	3.09 (2.33, 3.77)	-23.86	0.27 (0.26, 0.28)*	-1.05 (-1.24, -0.86)*
Oceania	9.28 (8.4, 10.36)	12.29 (11.05, 13.7)	24.49	6.94 (5.7, 8.46)	8.12 (6.42, 9.97)	14.46	0.97 (0.93, 1)*	0.51 (0.39, 0.63)*
Australasia	23.00 (21.35, 24.68)	26.23 (23.91, 28.56)	12.31	2.11 (1.69, 2.6)	2.96 (2.13, 3.94)	28.64	0.46 (0.4, 0.52)*	1.29 (0.9, 1.68)*
Eastern Europe	8.84 (7.99, 9.84)	12.66 (11.43, 14.07)	30.13	0.91 (0.75, 1.1)	1.17 (0.93, 1.42)	21.64	1.25 (1.18, 1.33)*	0.93 (0.43, 1.43)*
Western Europe	19.57 (17.83, 21.33)	21.73 (19.9, 23.55)	9.98	2.7 (2.16, 3.29)	3.44 (2.64, 4.24)	21.54	0.36 (0.35, 0.37)*	0.79 (0.5, 1.07)*
Central Europe	11.28 (10.16, 12.49)	17.8 (16.13, 19.58)	36.59	2.3 (1.92, 2.73)	2.44 (1.92, 3.07)	5.66	1.58 (1.56, 1.6)	0.19 (0, 0.39)
High-income North America	25.46 (23.25, 27.86)	27.26 (25, 29.56)	6.61	4.06 (3.39, 4.68)	6.6 (5.39, 7.69)	5.06	0.24 (0.21, 0.27)	1.71 (1.5, 1.91)
Andean Latin America	12.41 (11.24, 13.73)	23.54 (21.28, 25.83)	47.29	7.11 (5.8, 8.67)	10.6 (8.13, 13.49)	32.91	2.24 (2.2, 2.28)	1.43 (1.23, 1.63)
Central Latin America	21.15 (19.24, 23.43)	31.14 (28.98, 33.58)	32.08	6.98 (5.84, 8.22)	12.51 (9.94, 15.32)	44.21	1.34 (1.3, 1.38)	2.04 (1.84, 2.24)
Caribbean	12.33 (11.15, 13.66)	19.78 (18.06, 21.6)	37.69	4.76 (3.87, 5.73)	6.41 (5.08, 8.03)	25.69	1.66 (1.63, 1.69)	1.1 (0.88, 1.32)
Tropical Latin America	14.97 (13.57, 16.39)	20.09 (18.42, 22.03)	25.46	5.05 (4.2, 5.91)	5.32 (4.36, 6.35)	5.03	1.01 (0.97, 1.05)	0.22 (-0.07, 0.5)
Southern Latin America	18.55 (16.59, 20.46)	24.97 (22.76, 27.2)	25.70	7.24 (5.99, 8.54)	9.43 (7.7, 11.22)	23.24	1.03 (0.99, 1.06)	0.97 (0.69, 1.24)
Eastern Sub-Saharan Africa	7.62 (6.94, 8.29)	9.69 (8.78, 10.62)	21.34	9.99 (8.05, 12.14)	8.84 (7.33, 10.5)	-13.01	0.83 (0.8, 0.86)	-0.44 (-0.56, -0.31)
Southern Sub-Saharan Africa	13.5 (12.22, 14.89)	18.54 (16.85, 20.33)	27.20	8.79 (7.3, 10.78)	12.5 (10.57, 14.7)	29.67	1.1 (1.05, 1.14)	1.23 (0.72, 1.75)
Western Sub-Saharan Africa	10.52 (9.65, 11.5)	14.11 (12.87, 15.43)	25.43	13.18 (10.93, 16.04)	11.86 (9.78, 13.93)	-11.14	1.01 (0.94, 1.08)	-0.36 (-0.46, -0.25)
North Africa and Middle East	21.13 (19.25, 23.14)	36.55 (33.59, 39.58)	42.19	11.94 (9.6, 15.89)	10.6 (8.53, 12.82)	-12.70	1.91 (1.88, 1.95)	-0.4 (-0.62, -0.18)
Central Sub-Saharan Africa	7.36 (6.68, 8.09)	9.97 (8.98, 11.02)	26.25	11.02 (8.93, 13.39)	9.59 (7.04, 12.34)	-14.90	1.06 (1.03, 1.09)	-0.48 (-0.58, -0.38)

Results for SDI regions and Geographical Regions are for both sexes; *Statistically significant increase or decrease; ASIR, age-standardized incidence rate; ASDR, age-standardized deaths rate; AAPC, average annual percentage change; SDI, socio-demographic index. # Change of ASRs were calculated by 100 × (ASR2019-ASR1990)/ASR2019

For SDI regions, the high-SDI region had the highest ASIR (24.96 per 100k population; 95% CI, 23.02 to 27.05), while the low-SDI regions had a top ASDR with 8.16 per 100k population (95% CI, 6.85 to 9.66) (Table 1). The incidence and death burden of CKD due to hypertension burden in the 21 geographical regions were presented in Table 1, with the highest incidence burden were observed in the North Africa and Middle East with ASIRs were 36.55 (95% CI, 33.59 to 39.58) per 100k population, and the Central Latin America were observed the highest ASDRs (12.51; 95% CI, 9.9 to 15.32).

**Fig. 2:**
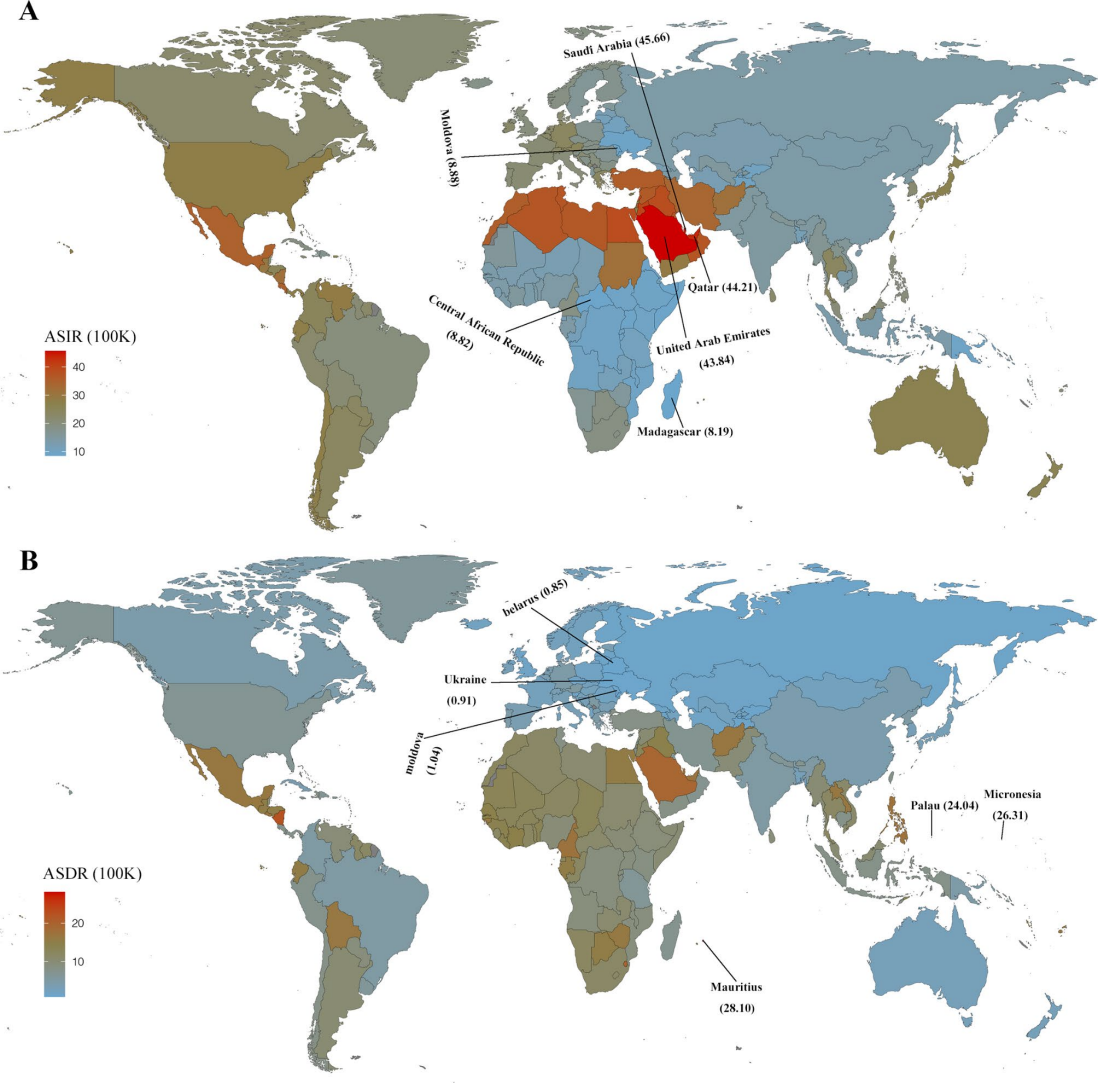
The age-standardized incidence rate (ASIR, A) and the age-standardized deaths rate (ASDR, B) of CKD due to hypertension for both sexes in 204 countries and territories in 2019

Regarding the 204 countries, the highest ASIR was observed in the Saudi Arabia (45.66 per 100k population; 95% CI, 41.39 to 50.22), followed by the Qatar and the United Arab Emirates (Fig. 2A, Supplementary Table 1). The lowest ASIR was observed in the Madagascar, followed by the Central African Republic, and Moldova (Fig. 2A, Supplementary Table 1). By ASDR, countries Mauritius was observed the highest rate by 28.10 per 100k population (95% CI, 21.91 to 35.16), and followed by, Micronesia and Palau (Fig. 2B, Supplementary Table 2). On the contrary, the top three lowest ASDRs were the Belarus, Ukraine and Moldova (Fig. 2, Supplementary Table 2).

### Incidence trends estimates of CKD due to hypertension from 1990 to 2019

**Fig. 3:**
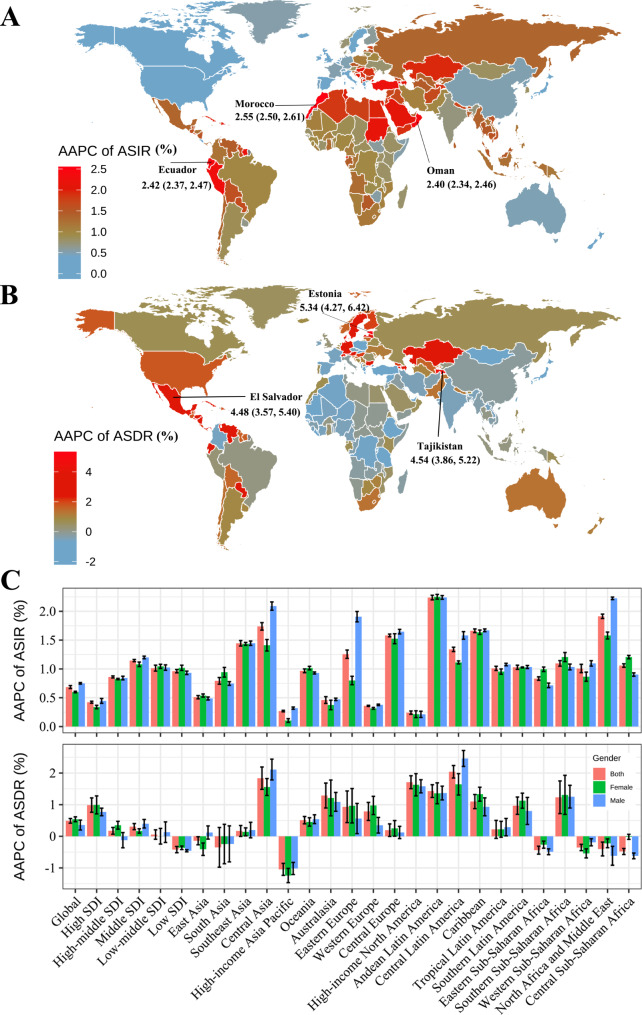
The annual variation of CKD due to hypertension burden from 1990 to 2019, by regions and countries. AAPCs of age-standardized incidence (ASIR, **A**) and deaths rates (ASDR, **B**) in 204 countries/territories. AAPCs of age-standardized incidence (**C**) and deaths rates (**D**) in SDI quintiles and 21 GBD geographic regions, by sex. AAPC, average annual percentage changes; SDI, social-demographic index

Globally, the ASIR of CKD due to hypertension increased, with an AAPC (%) of 0.69 (95%CI, 0.66 to 0.71), from 15.97 (95%CI, 14.6 to 17.4) in 1990 to 19.45 (95%CI, 17.85, 21.09) per 100 K population in 2019 (Table 1). The most pronounced increase country was observed in the Morocco (AAPC, 2.55%), and followed by Ecuador (AAPC, 2.42%) and Oman (AAPC, 2.40%) (Fig. 3A, Supplementary Table 1). All the countries were observed significant increased ASIRs (AAPC > 0) except for the Ireland, Greece and UK (Supplementary Table 1).

**Fig. 4:**
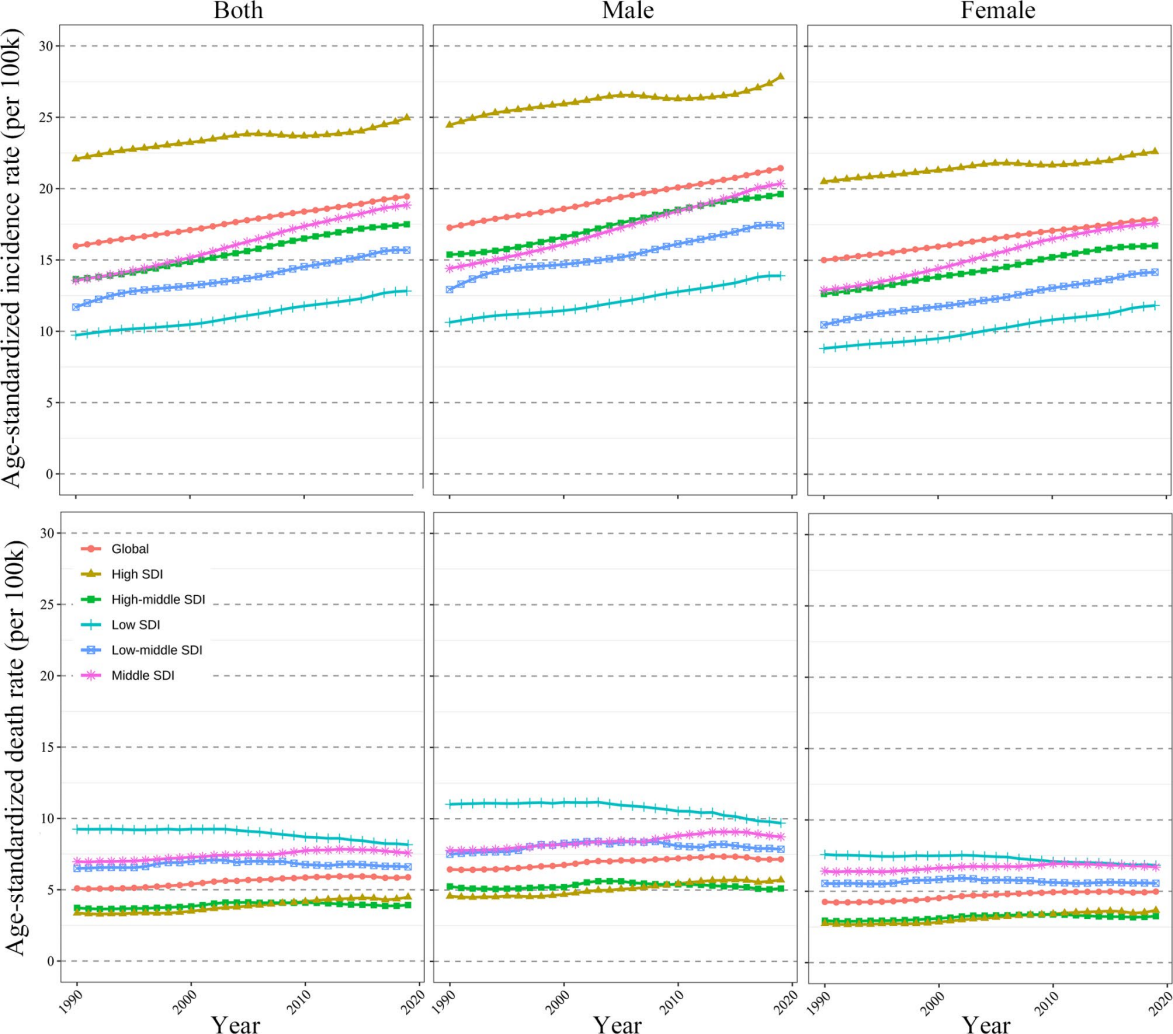
The age-standardized incidence rate (ASIR) and deaths rate (ASDR) of CKD due to hypertension, by SDI regions and gender from 1990 to 2019. SDI, social-demographic index

For regions, the ASIRs of the five SDI regions kept increasing from 1990 to 2019 (Fig. 4). The Middle SDI region was observed the lowest ASIR but highest percentage change (28.04%) and AAPC (1.15%; 95% CI, 1.13 to 1.16) (Table 1; Fig. 4). However, the high-SDI region was observed the highest ASIR by years and gender (Fig. 4), and the lowest percentage change (11.56%) and AAPC (0.42%; 95% CI, 0.41 to 0.44) (Table 1; Fig. 4). Similarly, the 21 geographical regions were all observed increased trends, with the highest region was the Andean Latin America (change percentage, 47.29%; AAPC 2.24, 95% CI, 2.20–2.28%) (Table 1). The gender differences of ASIRs change and AAPCs were shown in Fig. 3C and Supplementary Fig. 3.

### Deaths trends estimates of CKD due to hypertension from 1990 to 2019

The trends of ASDRs showed more heterogeneity than ASIRs around the world. Generally, the global ASDR increased from 1990 to 2019 by 13.29%, with an AAPC (%) of 0.49 (95%CI: 0.42, 0.56) (Table 1). According to our statistical analysis, compared with 1990, ASDRs in 2019 were observed significant increase among 127 countries (AAPC > 0), while 74 countries decreased (AAPC < 0), with the top 3 increased countries were the Estonia (AAPC, 5.34%), and followed by Tajikistan (AAPC, 4.54%) and EI Salvador (AAPC, 4.58%) (Fig. 3, Supplementary Table 2).

Regarding SDI regions, the low-SDI region was observed the highest but decreased ASDR from 1990 to 2019, with AAPC was − 0.42% (95% CI, -0.52% to -0.32%) (Table 1; Fig. 3D). The ASDRs slightly increased in the high-SDI, high-middle SDI, middle SDI, low-middle SDI regions with the AAPC range from 0.17 to 0.99% (Table 1; Fig. 4). Among the 21 geographical regions, ASDRs increased in 15 regions, including the greatest increase of the Central Latin America (change percentage = 44.22%; AAPC, 2.04%; 95% CI, 1.84–2.24%), followed by the high-income North America (AAPC, 1.71%; 95% CI, 1.50–1.91%) (Table 1). The global AAPC of ASDR in females was slightly higher than which in males (Table 1; Fig. 3D). Differently, lower AAPCs in females were observed in two SDI regions (middle SDI and low-middle SDI) and five geographical regions, including the Central Asia, Oceania, Andean Latin America, Central Latin America, and Tropical Latin America (Fig. 3D, Supplementary Fig. 5).

### Correlation between Burden of CKD due to hypertension and SDI

**Fig. 5:**
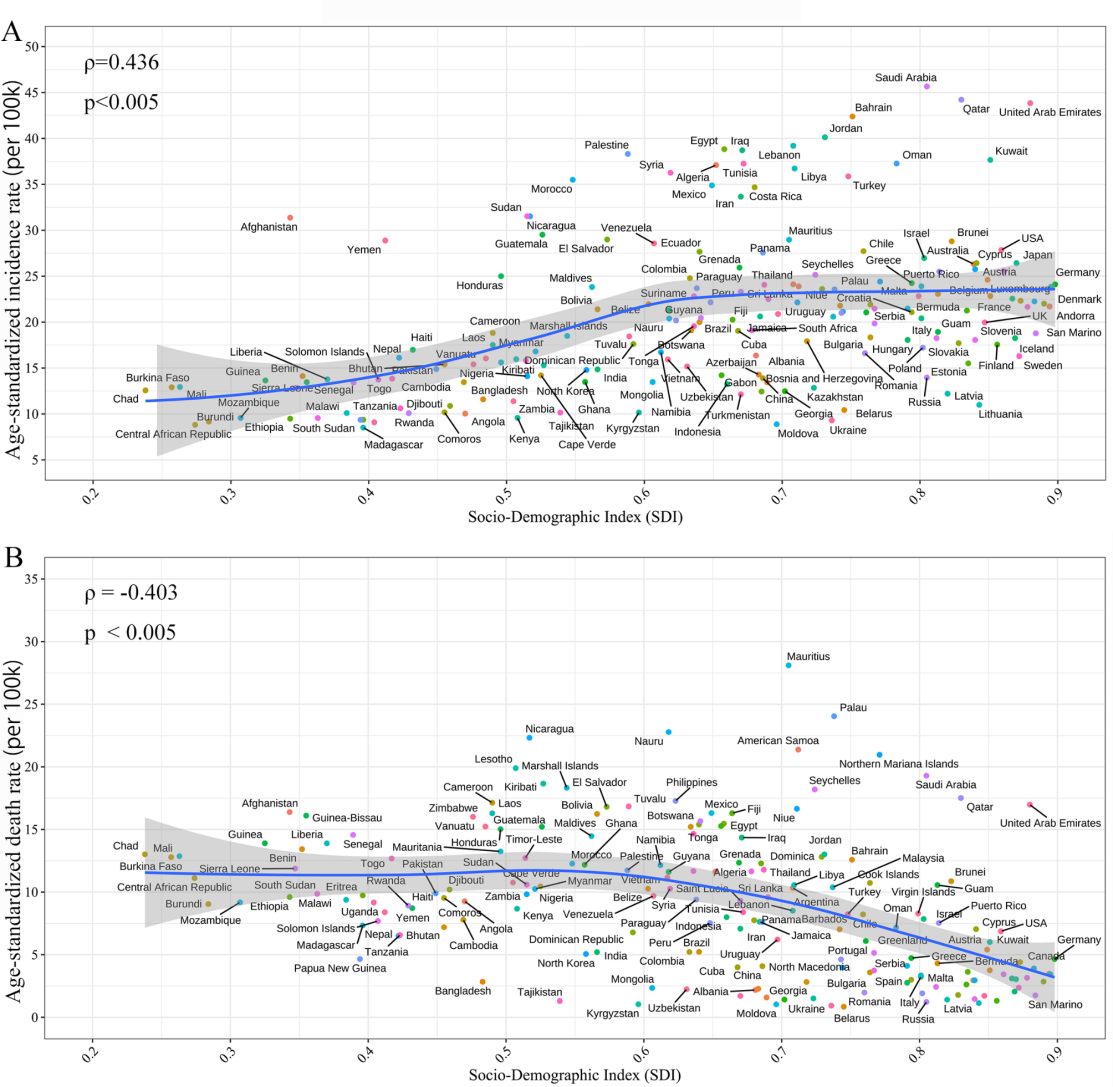
The correlation between SDI and ASR of CKD due to hypertension in 2019. (**A**) Correlation between SDI (in 2019) and age-standardized incidence rate (ASIR). (**B**) Correlation between SDI (in 2019) and age-standardized deaths rate (ASDR). SDI, social-demographic index

Supplementary Fig. 5 demonstrates the trend in ASIR and ASDR across SDI by geographical regions, from 1990 to 2019. ASIRs of regions generally followed the increasing incidence along with SDI (in 2019). However, decrease trend of ASDR corelated to SDI were observed in our results. Further correlations were analyzed between AAPCs of 204 countries and SDI (in 2019) (Fig. 5A). A significant positive association was found between AAPCs of ASIR and SDI (ρ = 0.22; P = 0.002) when the SDI was limited to below 0.63. For an SDI above 0.63, the association was observed as significant negative (ρ = -0.536; P < 0.001). Besides, a significant negative association (ρ = 0.503; P < 0.001) was observed between AAPCs of ASDRs and SDI (in 2019) (Fig. 5B). These results reveal that countries with middle SDI have experienced a heaviest increasing incidence burden, and lower SDI countries have experienced a more rapid increase in deaths burden of hypertension related CKD from 1990 to 2019.

### Spatial autocorrelation of CKD due to hypertension

**Fig. 6:**
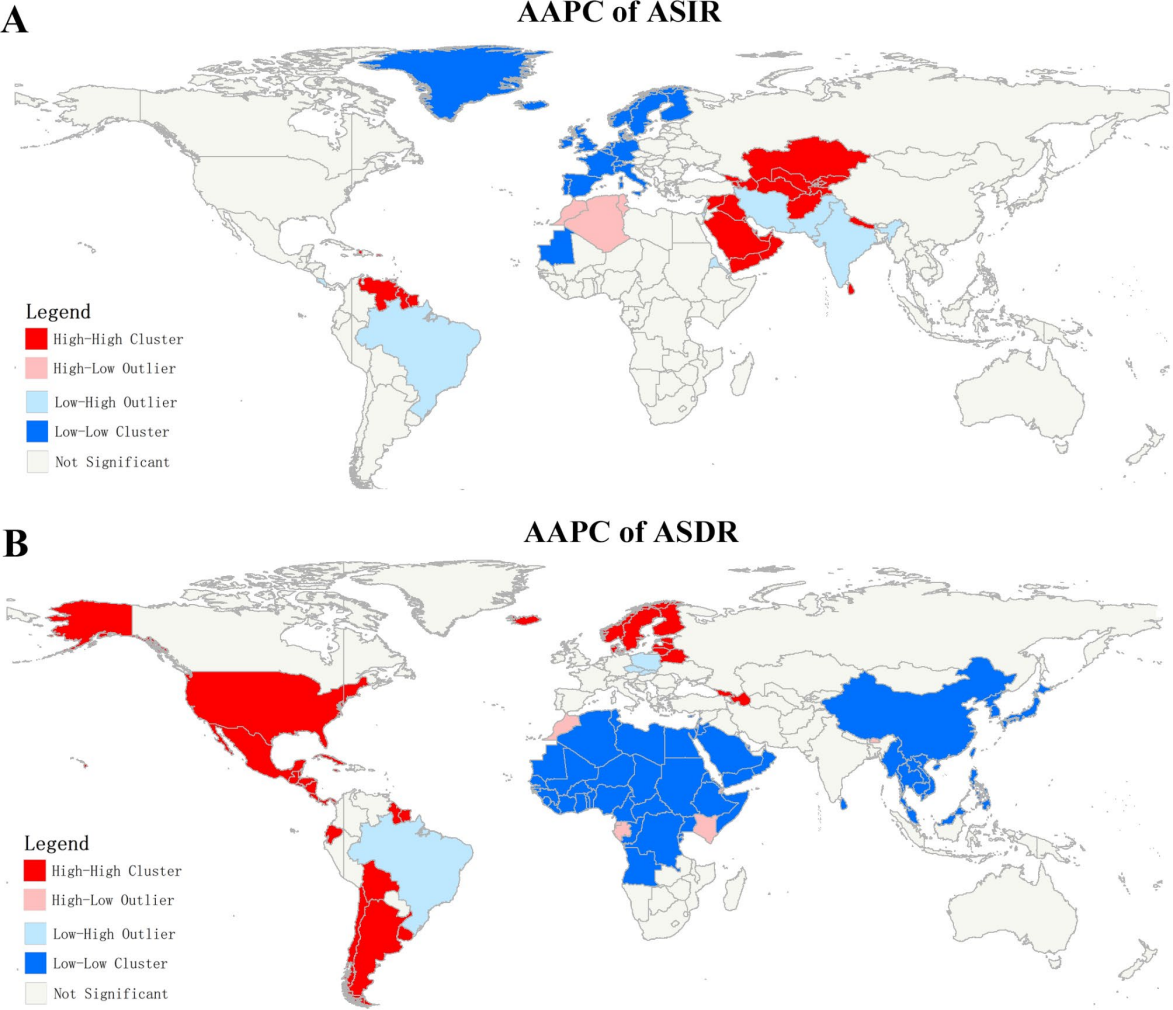
Regionalization of 204 countries based on the local index of spatial autocorrelation (LISA) analysis of CKD due to hypertension for age-standardized incidence (ASIR, **A**), and age-standardized death rate (ASDR, **C**) in 2019, and their average annual percentage changes (AAPCs) from 1990 to 2019 (**B**, **D**)

Spatially, the global autocorrelation analysis showed no significant spatial clustering pattern for the ASIR (Moran’s I, -0.007; Z, -0.07; p = 0.94) and ASDR (Moran’s I, 0.128; Z, 1.47; p = 0.14) of CKD due to hypertension (Supplementary Fig. 7A, 8A). However, the Moran’s diagram implies a positive spatial autocorrelation for the AAPC of ASIRs (Moran’s I, 0.191; Z, 8.66; p < 0.001) from 1990 to 2019, with 19 countries (Kazakhstan, Uzbekistan, Afghanistan, Iraq, Saudi Arabia, United Arab Emirates, et al.) were found in the high-high quadrant (Fig. 6A). Meanwhile, positive spatial autocorrelation for the AAPC of ASDRs (Moran’s I, 0.254; Z, -0.07; p < 0.001) were observed, and 21 countries (including US, Mexico, Argentina, Chile, Bolivia, Norway, Sweden, Finland, et al.) were clustered into high-high quadrant (Fig. 6B).

## Discussion

In response to our research objectives and hypothesis, this study conducted a comprehensive analysis of the global, regional, and national burden and trends of hypertension-related CKD from 1990 to 2019, yielding several key findings. First, we confirmed a persistent increase in both ASIR and ASDR of CKD attributed to hypertension on a global scale. Second, Middle-developed regions, such as Andean Latin America, North Africa and the Middle East, and Central Asia, exhibited the highest increasing ASIR trends, while High-developed regions, including High-income North America and Central Latin America, showed the highest increasing ASDR trends. These results underscore the regional disparities in CKD burden. Third, our analysis revealed greater complexity in ASDR trends across global regions and countries compared to ASIR trends. Fourth, our study highlighted that hypertension-related CKD disproportionately affects males and the elderly population, emphasizing the need for targeted interventions in these demographic groups. Fifth, spatial autocorrelation analysis demonstrated significant positive spatial autocorrelation for the AAPC of both ASDRs and ASIRs from 1990 to 2019. These findings provide insights into the spatial distribution and clustering of hypertension-related CKD trends, further informing effective prevention and control strategies.

Global trends from 1990 to 2019 show increasing ASIR and ASDR of CKD due to hypertension, which is similar to the trend of CKD due to type 2 diabetes [19]. The increase trend of ASDR of CKD due to hypertension was consist with the ASDR of CKD and resulted in the number of deaths caused by CKD to rise from 17th to 12th in the globally [1]. However, in the past 30 years, the global ASDR for cardiovascular disease, cancer, and COPD decreased by 30.4%, 14.9%, and 41.4%, respectively [20]. These findings underscore the need for greater attention to be given to global healthcare resources and policies in the treatment of highly prevalent chronic conditions, including CKD, and hypertension-related kidney disease.

Hypertension and related chronic conditions such as cardiovascular diseases were widely observed on a global scale, but regional variations were pronounced [20, 21]. Hypertension-related CKD burden also discrepancies at SDI, regional, and national. High-SDI quintile regions the lowest ASDR and increasing trend of ASIR. These results suggest that, from 1990 to 2019, high-SDI regions effectively controlled hypertension-related CKD. This may be associated with the ongoing improvement of diagnosis, medical care, blood pressure management and treatment in these regions [22–24]. The most pronounced increase of ASIR was observed in middle-SDI quintile. From 1990 to 2019, a number of locations, notably Morocco, Ecuador, Oman, Peru, Turkey, and Armenia, experienced significant increases in both proportion and AAPCs. These can be explained by many driven factors, including organizational policy and awareness lacking, degree of population aging changing, lifestyle and diet adjustment according to the economic development, which had gone through by the high-SDI region [25]. Low-SDI region had the highest ASDR, which may be due to the lack of access to renal replacement therapy [26].

The ASIR and ASDR were in reverse correlation to SDI in 2019, that means low-developed countries in the low-SDI quintile was bearing the heaviest burden of fatality, and the highly developed quintile was bearing the heaviest incidence burden. This association may also be explained by dietary structure, physical health, well-established medical systems, and popularization of low-cost treatment for both hypertension and CKD [27–29]. This phenomenon suggests that early layout prevention measures are needed in high-SDI quintile countries and the allocation of medical resources could be tilted to low-SDI countries. A notable situation is, from 1990 to 2019, all regions observed an increasing trend of ASIR following the increasing SDI, however, an asymmetrically inverted V-shaped correlation between AAPCs and SDI. This finding reveals that nations in the middle quintile of SDI bore the heaviest weight of increasing incidence, revealing the history of high-SDI countries and the future of low-SDI countries.

In 2019, elderly individuals vastly surpassed young people in absolute incidence and fatal load, and the situation was comparable for both sexes. Our data supported the concept that women were more resistant to hypertension-related CKD than men. The incidence rate and deaths rate in males were higher than which in females by all age-periods. Similar sex broken distribution in age-periods were observed in the SDI regions. Sex preponderance was unquestionably present in hypertension-related CKD, as it was in other hypertension-related conditions, such as cardiovascular diseases (CVDs) [30]. It was discovered that women have higher proportions of hypertension awareness, treatment and control than men [27]. In fact, differences in behavioral and metabolic risk variables substantially account for the gender disparity in hypertension among men [31]. There were evidences of substantial gender disparities in access to CKD treatment [32]. Studies also shown that inherent sex heterogeneity in CKD, end-stage kidney disease and CVDs in younger women compared to men, and omitting this gender protection after menopause, which was demonstrated as the outcome of estrogen exhausted [33]. However, our data shows some special performances that attract attention. First, elderly theoretically postmenopausal females, without protection with estrogen, were still lower in ASIR and ASDR which need more studies to clarify the complex risk factors. Second, the estimated annual percentage changes of ASDR reveal a global upward tendency, particularly in those locations with a median SDI. This clinically significant difference between the sexes necessitates heightened awareness and the adoption of sex-specific measures to ensure equal access to kidney health care for the purpose of enhancing prevention and treatment.

In order to investigate, analyze, and explain spatial patterns of CKD due to hypertension, we have applied the techniques of spatial autocorrelation, both global and local methods. We assessed the spatial autocorrelation of CKD due to hypertension, and identified significant spatial clusters of AAPCs of ASIR and ASDR, with relatively large geographical difference. The results of the local indices show the existence of significant high to high clusters countries of AAPC of ASIR were located in Middle or Low-middle SDI regions, while the significant high to high clusters countries of AAPC of ASDR were located in High SDI regions. These results are likely to indicate the presence of common predisposing factors in these regions, such as economy, education, lifestyle habits, dietary habits. Although our study does not further elucidate the specific causes of spatial clustering, the findings of this research may have potential applications in assisting relevant authorities in making decisions regarding policies and regulations for the control and prevention of CKD due to hypertension.

The worldwide prevalence of CKD is on a steep incline and is projected to rank as the fifth leading cause of global mortality by 2040 [34]. As hypertension stands out as a primary risk factor for CKD development, underscoring the significance of mitigating the CKD burden attributable to hypertension in the broader effort to diminish CKD’s overall impact. Thus, our results could provide a clear understanding the distribution of the global burden of CKD due to hypertension, and contributed for public health prioritization, guiding preventative strategies, addressing health disparities, fostering research and innovation, and assessing the impact of interventions, as well as enables effective resource allocation, reduces health inequalities, and informs the development of better diagnostics and treatments.

However, this study has several limitations. First, A 2021 article reported on the global burden of hypertensive kidney disease from 1990 to 2019 using DALYs, which somewhat reduced the novelty of our study [35]. However, in contrast to previous work, we focused on using incidence and death rates as indicators, which may better reflect the immediate control and treatment benefits for hypertensive kidney disease. Additionally, in our study, we employed spatial autocorrelation analysis to identify regional clustering of changes in incidence and mortality rates, which could potentially provide richer information for policy-making and the development of prevention and treatment strategies. Second, the major limitation is the integrity and accuracy of data from the GBD database, which were estimated from mathematical models based on surveillance data rather than surveillance data itself [1, 11, 36]. Third, there are results bias risks as GBD 2019 adjusted its data sources, collation, and analytical strategies to decrease missing data and improve its data quality and comparability. Fourth, despite the GBD study followed the standardized definition of CKD presented by the KDIGO guidelines, sources of information on non-fatal CKD were influenced by many factors (such as sampling, laboratory methods, and the equation used to calculate eGFR) [37]. Meanwhile, Different global hypertension diagnostic methods and CKD standards can impact condition estimates. GBD study algorithm changes during evaluation increase uncertainty due to limited data sources and potential bias in demographic subgroups. Fifth, global, regional and country levels were analyzed in our study, however, further analyzing discrepancies in domestic areas were absence. Last but not least, the risk factors and clinical information of hypertension-related CKD cannot be available in GBD database, therefore the causes for the shifting patterns in burden should be further examined.

## Conclusion

This study conducted comprehensive analyses of the incidence and mortality burden of hypertension-related CKD. Our findings indicate high-SDI, and low SDI regions and countries require more efficient strategies and increased investment in the prevention and control of the escalating mortality burden associated with CKD due to hypertension. Additionally, our research highlights the importance of prioritizing the elderly population and males in healthcare interventions, and emphasizing the importance of focusing on preventive measures for the younger population. Overall, these results underscore the need for targeted efforts and resource allocation based on regional variations and population demographics to effectively mitigate the burden of CKD due to hypertension.

### Electronic supplementary material

Below is the link to the electronic supplementary material.

Supplementary Material 1

## Data Availability

The datasets generated and analysed during the current study are available in the official website of the GBD 2019 Study (http://ghdx.healthdata.org), and download according to the Global Health Data Exchange (GHDx) query tool (http://ghdx.healthdata.org/gbd-results-tool).
