# Correction: Global incidence and death estimates of chronic kidney disease due to hypertension from 1990 to 2019, an ecological analysis of the global burden of diseases 2019 study

**DOI:** 10.1186/s12882-023-03452-3

**Published:** 2024-01-04

**Authors:** Yan Liu, Qin He, Qiying Li, Min Tian, Xiaojiao Li, Xufei Yao, Dongmei He, Chunying Deng

**Affiliations:** 1 Department of Nephrology, Chengdu Second People’s Hospital, No. 2 Huatai Road, 610000 Chenghua District, Chengdu, Sichuan Province China; 2 Department of Endocrine, The fourth people’s hospital of Zi Gong, No. 400, North Dangui Street, 643000 Ziliujing District, Zigong, Sichuan Province China; 3 Department of Stomatology, Traditional Chinese Medicine Hospital, No. 800 Zhongshan Street, 610000 Lishui City, Zhejiang Province China

**Correction: BMC Nephrology (2023) 24:352**

10.1186/s12882-023-03391-z

Following publication of the original article [**1**], the authors identified an error in the author name of Xufei Yao.

The incorrect author name is: Xufeng Yao.

The correct author name is: Xufei Yao.
