# Supplementary material for: Global incidence and death estimates of chronic kidney disease due to hypertension from 1990 to 2019, an ecological analysis of the global burden of diseases 2019 study
Source: BMC Nephrol. 2023 Nov 29;24:352. doi: 10.1186/s12882-023-03391-z (PMC10687895; doi:10.1186/s12882-023-03391-z)
Supplement: Supplementary file 1 — Supplementary Material 1 [file 12882_2023_3391_MOESM1_ESM.docx]

**Global incidence and death estimates of chronic kidney disease due to hypertension from 1990 to 2019, an ecological analysis of the Global Burden of Diseases 2019 study**

Yan Liu^1^, Qin He^1^, Qiying Li^2^, Min Tian^1^, Xiaojiao, Li^2^, Xufeng, Yao^3^, Dongmei He^1#^, Chunying Deng^2#^

^1^ Department of Nephrology, Chengdu Second People’s Hospital, Chengdu, China.

^2^ Department of Endocrine, the fourth people’s hospital of Zi Gong, Zi Gong, China.

^3^ Department of Stomatology, Traditional Chinese Medicine Hospital, Lishui City, Zhejiang Province, china.

**#Correspondence:**

Xufeng Yao, No. 800 Zhongshan Street, Lishui City, Zhejiang Province，China. Email: 13600606060@139.com.
Dongmei He, No. 2 Huatai Road, Chenghua District, Chengdu City, Sichuan Province, China, 610000. Email: [Hdmlmy@163.com](mailto:Hdmlmy@163.com).

Chunying Deng, No. 400, North Dangui Street, Ziliujing District, Zigong City, Sichuan Province, China, 643000. Email: cy_deng@163.c

**Supplementary table 1.** Trend of age-standardized incidence rate (ASIR) from 1990 to 2019 in the 204 countries.

| location | ASIR 1990  ($\times100, 000$) | ASIR 2019  ($\times100, 000$) | Change percentage  (%)# | AAPCs  (%)* |
| --- | --- | --- | --- | --- |
| Afghanistan | 19.44 (17.12, 21.94) | 31.37 (27.72, 35.4) | 38.02 | 1.67(1.66, 1.69) |
| Albania | 10.14 (8.84, 11.59) | 16.36 (14.33, 18.57) | 38.05 | 1.66(1.62, 1.69) |
| Algeria | 21.9 (19.51, 24.63) | 37.09 (33.28, 41.16) | 40.96 | 1.84(1.81, 1.87) |
| American Samoa | 16.72 (14.74, 19.05) | 23.89 (21.15, 27.12) | 30.02 | 1.23(1.19, 1.28) |
| Andorra | 20.89 (18.64, 23.63) | 21.71 (19.37, 24.32) | 3.74 | 0.13(0.1, 0.16) |
| Angola | 6.94 (6.15, 7.76) | 10.03 (8.83, 11.37) | 30.79 | 1.28(1.23, 1.32) |
| Antigua | 15.99 (14.05, 17.9) | 24.7 (22.03, 27.63) | 35.24 | 1.5(1.49, 1.52) |
| Argentina | 18.68 (16.54, 20.81) | 24.12 (21.72, 26.73) | 22.57 | 0.88(0.86, 0.91) |
| Armenia | 7.21 (6.18, 8.49) | 13.46 (11.72, 15.5) | 46.45 | 2.19(2.02, 2.37) |
| Australia | 22.93 (21.23, 24.68) | 26.32 (23.85, 28.84) | 12.88 | 0.48(0.41, 0.56) |
| Austria | 19.43 (17.26, 21.71) | 24.61 (21.81, 27.29) | 21.06 | 0.82(0.79, 0.84) |
| Azerbaijan | 7.89 (6.73, 9.19) | 14.29 (12.32, 16.34) | 44.8 | 2.08(1.87, 2.29) |
| Bahamas | 14.4 (12.64, 16.25) | 21.09 (18.56, 23.73) | 31.73 | 1.32(1.29, 1.35) |
| Bahrain | 25.47 (22.9, 28.43) | 42.39 (38.27, 46.81) | 39.92 | 1.78(1.75, 1.8) |
| Bangladesh | 7.63 (6.67, 8.62) | 11.59 (10.2, 13.22) | 34.17 | 1.44(1.41, 1.48) |
| Barbados | 14.18 (12.41, 16.07) | 21.79 (19.18, 24.53) | 34.93 | 1.49(1.47, 1.51) |
| Belarus | 7.81 (6.72, 9.01) | 10.43 (9.02, 11.93) | 25.08 | 1.01(0.91, 1.11) |
| Belgium | 21.03 (18.85, 23.33) | 22.84 (20.7, 25.28) | 7.93 | 0.29(0.25, 0.32) |
| Belize | 14.07 (12.33, 16.11) | 21.95 (19.49, 24.73) | 35.91 | 1.54(1.49, 1.58) |
| Benin | 10.61 (9.51, 11.93) | 14.12 (12.47, 15.94) | 24.88 | 0.98(0.96, 1.01) |
| Bermuda | 14.23 (12.43, 16.02) | 23.07 (20.36, 25.88) | 38.31 | 1.68(1.63, 1.73) |
| Bhutan | 9.65 (8.52, 10.95) | 15.39 (13.55, 17.55) | 37.29 | 1.63(1.58, 1.67) |
| Bolivia | 13.53 (12.1, 15.19) | 21.4 (19.1, 23.68) | 36.76 | 1.6(1.58, 1.63) |
| Bosnia and Herzegovina | 10.01 (8.84, 11.33) | 17.92 (15.86, 20.09) | 44.16 | 2.07(1.98, 2.16) |
| Botswana | 12.45 (10.93, 14.12) | 19.11 (16.84, 21.66) | 34.82 | 1.48(1.42, 1.53) |
| Brazil | 14.98 (13.57, 16.4) | 20 (18.34, 21.9) | 25.09 | 0.99(0.95, 1.03) |
| Brunei | 26.64 (23.67, 29.52) | 28.8 (25.49, 32.18) | 7.49 | 0.27(0.23, 0.31) |
| Bulgaria | 11.2 (9.77, 12.83) | 18.34 (16.09, 20.74) | 38.93 | 1.72(1.69, 1.75) |
| Burkina Faso | 9.47 (8.51, 10.56) | 12.91 (11.45, 14.5) | 26.68 | 1.08(1.05, 1.11) |
| Burundi | 7.4 (6.64, 8.21) | 9.17 (8.18, 10.28) | 19.31 | 0.75(0.71, 0.78) |
| Cambodia | 8.57 (7.5, 9.71) | 13.46 (11.91, 15.35) | 36.35 | 1.66(1.61, 1.71) |
| Cameroon | 13.67 (12.24, 15.24) | 18.82 (16.66, 21.16) | 27.37 | 1.56(1.53, 1.59) |
| Canada | 21.92 (19.48, 24.17) | 22.32 (20.07, 24.83) | 1.77 | 1.11(1.06, 1.17) |
| Cape Verde | 8.83 (7.82, 10) | 14.2 (12.5, 16.22) | 37.79 | 0.06(-0.02, 0.14) |
| Central African Republic | 6.91 (6.2, 7.74) | 8.82 (7.81, 9.87) | 21.64 | 0.85(0.82, 0.88) |
| Chad | 9.85 (8.79, 11.01) | 12.57 (11.12, 14.23) | 21.6 | 0.84(0.8, 0.87) |
| Chile | 18.7 (16.68, 20.9) | 27.73 (24.86, 30.55) | 32.55 | 1.37(1.34, 1.39) |
| China | 12.01 (10.77, 13.38) | 13.89 (12.59, 15.31) | 13.55 | 0.51(0.48, 0.53) |
| Colombia | 17.41 (15.26, 19.76) | 24.78 (22.05, 27.93) | 29.73 | 1.22(1.18, 1.26) |
| Comoros | 7.9 (7.1, 8.78) | 10.17 (9.03, 11.37) | 22.32 | 0.87(0.83, 0.92) |
| Cook Islands | 14.42 (12.76, 16.2) | 21.92 (19.31, 24.72) | 34.2 | 1.2(1.14, 1.27) |
| Costa Rica | 33.78 (31.06, 36.7) | 34.69 (31.87, 37.79) | 2.62 | 1.44(1.41, 1.48) |
| Croatia | 13.19 (11.46, 14.88) | 21.08 (18.62, 23.68) | 37.44 | 0.09(0.09, 0.1) |
| Cuba | 10.8 (9.4, 12.37) | 19.05 (16.89, 21.48) | 43.31 | 0.79(0.74, 0.85) |
| Cyprus | 24.99 (22.38, 27.77) | 26.42 (23.56, 29.48) | 5.41 | 1.64(1.59, 1.69) |
| Czech Republic | 11.39 (10.01, 12.92) | 17.71 (15.59, 19.99) | 35.67 | 1.98(1.93, 2.04) |
| Democratic Republic of the Congo | 7.37 (6.63, 8.18) | 9.76 (8.64, 10.88) | 24.48 | 0.18(0.1, 0.25) |
| Denmark | 17.87 (15.86, 20.07) | 22.01 (19.78, 24.48) | 18.78 | 1.53(1.48, 1.57) |
| Djibouti | 7.96 (7.13, 8.87) | 10.87 (9.79, 12.17) | 26.84 | 0.82(0.71, 0.92) |
| Dominica | 17.2 (15.22, 19.43) | 23.6 (20.96, 26.58) | 27.14 | 0.98(0.94, 1.01) |
| Dominican Republic | 9.86 (8.62, 11.27) | 17.61 (15.49, 19.84) | 44.04 | 0.72(0.71, 0.74) |
| Ecuador | 13.8 (12.15, 15.65) | 27.66 (24.81, 30.64) | 50.12 | 1.09(1.03, 1.14) |
| Egypt | 22.11 (19.48, 24.64) | 38.83 (34.52, 43.1) | 43.06 | 1.09(1.06, 1.13) |
| El Salvador | 17.46 (15.24, 20.11) | 28.99 (25.65, 32.91) | 39.77 | 2.04(1.94, 2.14) |
| Equatorial Guinea | 6.91 (6.19, 7.62) | 12.44 (11.08, 13.84) | 44.47 | 2.42(2.37, 2.47) |
| Eritrea | 6.76 (5.99, 7.54) | 9.36 (8.28, 10.52) | 27.86 | 1.97(1.94, 2) |
| Estonia | 9.38 (8.11, 10.98) | 15.51 (13.49, 17.91) | 39.53 | 1.77(1.74, 1.8) |
| Ethiopia | 7.49 (6.73, 8.21) | 9.48 (8.46, 10.49) | 21.08 | 2.06(2, 2.12) |
| Fiji | 15.48 (13.64, 17.52) | 20.26 (18.02, 22.75) | 23.61 | 1.14(1.11, 1.17) |
| Finland | 14.7 (12.78, 16.76) | 17.55 (15.7, 19.63) | 16.25 | 1.76(1.71, 1.82) |
| France | 18.29 (16.38, 20.41) | 21.26 (19.05, 23.6) | 13.96 | 0.9(0.85, 0.96) |
| Gabon | 9.34 (8.39, 10.41) | 14.21 (12.61, 15.76) | 34.25 | 0.82(0.8, 0.84) |
| Gambia | 10.4 (9.3, 11.7) | 13.86 (12.32, 15.7) | 24.97 | 0.92(0.89, 0.96) |
| Georgia | 7.84 (6.75, 9.15) | 12.48 (10.78, 14.28) | 37.16 | 0.6(0.58, 0.62) |
| Germany | 20.86 (18.63, 23.24) | 24.11 (22.04, 26.29) | 13.48 | 0.52(0.5, 0.53) |
| Ghana | 9.25 (8.17, 10.44) | 13.48 (11.93, 15.18) | 31.42 | 1.45(1.42, 1.48) |
| Greece | 24.54 (21.76, 27.24) | 24.25 (21.67, 26.9) | -1.23 | 0.99(0.97, 1.01) |
| Greenland | 18.05 (16.14, 20.22) | 21.06 (18.99, 23.5) | 14.28 | 1.63(1.58, 1.67) |
| Grenada | 15.65 (13.87, 17.69) | 25.93 (23.08, 29.09) | 39.65 | 0.5(0.49, 0.52) |
| Guam | 13.92 (12.19, 15.81) | 18.93 (16.64, 21.44) | 26.47 | 1.31(1.28, 1.34) |
| Guatemala | 18.7 (16.48, 21.4) | 29.52 (26.13, 33.24) | 36.66 | -0.04(-0.09, 0.01) |
| Guinea | 10.36 (9.25, 11.56) | 13.64 (12.03, 15.32) | 24.04 | 0.53(0.52, 0.54) |
| Guinea-Bissau | 11.02 (9.86, 12.38) | 13.46 (11.93, 15.28) | 18.09 | 1.75(1.74, 1.76) |
| Guyana | 13.19 (11.69, 14.84) | 21.33 (18.91, 23.89) | 38.14 | 1.06(1.04, 1.09) |
| Haiti | 11.69 (10.23, 13.27) | 16.99 (14.98, 19.21) | 31.17 | 1.6(1.55, 1.65) |
| Honduras | 17.31 (15.19, 20.06) | 25 (22.05, 28.62) | 30.76 | 0.95(0.93, 0.97) |
| Hungary | 9.77 (8.56, 11.11) | 18.06 (15.94, 20.41) | 45.9 | 0.69(0.66, 0.72) |
| Iceland | 16.85 (15.04, 18.92) | 18.25 (16.21, 20.49) | 7.72 | 1.67(1.62, 1.72) |
| India | 12.14 (11, 13.36) | 14.85 (13.42, 16.33) | 18.27 | 1.3(1.28, 1.31) |
| Indonesia | 9.24 (8.34, 10.22) | 13.26 (11.96, 14.66) | 30.31 | 1.27(1.23, 1.32) |
| Iran | 24.85 (22.55, 27.39) | 33.66 (30.87, 36.58) | 26.17 | 2.15(2.11, 2.18) |
| Iraq | 23.82 (21.23, 26.73) | 38.71 (35.07, 42.91) | 38.47 | 0.26(0.22, 0.3) |
| Ireland | 23.48 (21.34, 25.44) | 22.56 (21.27, 23.79) | -4.09 | 0.71(0.65, 0.77) |
| Israel | 24.62 (21.97, 27.34) | 26.98 (24.23, 30.02) | 8.74 | 1.24(1.2, 1.28) |
| Italy | 19.21 (17.28, 21.3) | 20.41 (18.48, 22.55) | 5.91 | 1.06(1.04, 1.08) |
| Ivory Coast | 12.02 (10.8, 13.25) | 15.15 (13.53, 16.83) | 20.64 | 1.7(1.67, 1.73) |
| Jamaica | 15.1 (13.27, 17.16) | 20.62 (18.2, 23.39) | 26.76 | -0.14(-0.16, -0.12) |
| Japan | 24.02 (22.08, 26.16) | 26.43 (24.24, 28.84) | 9.14 | 0.31(0.28, 0.34) |
| Jordan | 23.61 (21.1, 26.2) | 40.13 (36.14, 44.36) | 41.17 | 0.21(0.18, 0.23) |
| Kazakhstan | 7.37 (6.28, 8.62) | 12.85 (11.1, 14.78) | 42.63 | 1.08(1.01, 1.15) |
| Kenya | 7.61 (6.9, 8.39) | 9.57 (8.63, 10.63) | 20.49 | 0.32(0.27, 0.38) |
| Kiribati | 10.93 (9.46, 12.59) | 15.31 (13.39, 17.47) | 28.63 | 1.85(1.82, 1.88) |
| Kuwait | 24.52 (21.82, 27.67) | 37.66 (33.53, 41.9) | 34.89 | 1.94(1.79, 2.1) |
| Kyrgyzstan | 6.93 (5.85, 8.33) | 10.16 (8.6, 12.01) | 31.76 | 0.79(0.75, 0.82) |
| Laos | 12.28 (10.85, 13.82) | 17.51 (15.52, 19.6) | 29.87 | 1.18(1.14, 1.23) |
| Latvia | 7.97 (6.83, 9.17) | 12.21 (10.57, 14.09) | 34.75 | 1.49(1.46, 1.52) |
| Lebanon | 21.83 (19.34, 24.59) | 39.2 (35.08, 43.45) | 44.3 | 1.36(1.28, 1.43) |
| Lesotho | 12.02 (10.67, 13.47) | 15.97 (14.19, 17.88) | 24.72 | 1.22(1.18, 1.27) |
| Liberia | 10.14 (9, 11.37) | 13.77 (12.15, 15.54) | 26.34 | 1.49(1.38, 1.59) |
| Libya | 22.02 (19.53, 24.6) | 36.73 (32.68, 40.75) | 40.04 | 2.05(1.99, 2.11) |
| Lithuania | 8.64 (7.45, 9.9) | 10.99 (9.62, 12.52) | 21.4 | 0.98(0.91, 1.05) |
| Luxembourg | 21.18 (18.99, 23.42) | 23.84 (21.34, 26.23) | 11.16 | 1.05(0.99, 1.11) |
| Madagascar | 6.78 (6, 7.63) | 8.51 (7.59, 9.57) | 20.39 | 1.78(1.76, 1.79) |
| Malawi | 7.93 (7.11, 8.85) | 10.1 (9.02, 11.28) | 21.47 | 0.86(0.8, 0.91) |
| Malaysia | 13.4 (11.92, 14.99) | 20.59 (18.37, 23.08) | 34.91 | 0.41(0.39, 0.42) |
| Maldives | 15.44 (13.49, 17.44) | 23.82 (21.01, 27.1) | 35.2 | 0.79(0.76, 0.82) |
| Mali | 9.7 (8.11, 11.66) | 12.94 (10.81, 15.86) | 25.03 | 0.84(0.77, 0.9) |
| Malta | 22.18 (19.85, 24.77) | 23.9 (21.3, 26.6) | 7.22 | 1.49(1.45, 1.52) |
| Marshall Islands | 12.67 (11.17, 14.46) | 18.49 (16.44, 20.88) | 31.5 | 1.5(1.46, 1.54) |
| Mauritania | 11.44 (10.15, 12.86) | 15.62 (13.77, 17.6) | 26.74 | 1.02(0.97, 1.07) |
| Mauritius | 19.04 (16.55, 21.5) | 28.97 (25.01, 32.57) | 34.27 | 0.25(0.22, 0.28) |
| Mexico | 23.51 (21.19, 26.18) | 34.89 (32.44, 37.52) | 32.6 | 1.31(1.29, 1.33) |
| Micronesia | 14.35 (12.4, 16.35) | 21.9 (19.21, 24.99) | 34.46 | 1.07(1.04, 1.11) |
| Moldova | 6.25 (5.44, 7.17) | 8.88 (7.62, 10.34) | 29.64 | 1.45(1.41, 1.48) |
| Monaco | 18.5 (16.4, 20.77) | 20.87 (18.59, 23.25) | 11.35 | 1.36(1.33, 1.39) |
| Mongolia | 10.57 (9.07, 12.33) | 13.47 (11.62, 15.47) | 21.53 | 1.46(1.35, 1.57) |
| Montenegro | 15.02 (13.18, 16.98) | 21.48 (18.94, 24.24) | 30.08 | 0.42(0.4, 0.43) |
| Morocco | 17.14 (14.99, 19.37) | 35.5 (31.42, 39.6) | 51.71 | 0.84(0.72, 0.95) |
| Mozambique | 7.07 (6.33, 7.83) | 9.56 (8.47, 10.7) | 26.13 | 1.25(1.22, 1.27) |
| Myanmar | 11.22 (9.8, 12.71) | 16.8 (14.84, 19.05) | 33.2 | 2.55(2.5, 2.61) |
| Namibia | 12.17 (10.82, 13.72) | 16.73 (14.74, 18.91) | 27.22 | 1.05(1.03, 1.08) |
| Nauru | 14.69 (12.82, 16.83) | 20.39 (17.94, 23.22) | 27.97 | 1.4(1.36, 1.44) |
| Nepal | 9.78 (8.57, 11.26) | 16.12 (14.11, 18.32) | 39.29 | 1.1(1.08, 1.13) |
| Netherlands | 19.7 (17.97, 21.51) | 22.26 (19.95, 24.58) | 11.49 | 1.14(1.12, 1.16) |
| New Zealand | 23.36 (20.93, 25.9) | 25.75 (23.13, 28.58) | 9.31 | 1.74(1.68, 1.79) |
| Nicaragua | 20.18 (17.41, 23.31) | 31.52 (27.73, 35.75) | 35.98 | 0.41(0.32, 0.5) |
| Niger | 9.59 (8.54, 10.71) | 12.43 (11.02, 14) | 22.88 | 0.34(0.33, 0.36) |
| Nigeria | 10.55 (9.59, 11.61) | 14.1 (12.72, 15.6) | 25.17 | 1.54(1.48, 1.6) |
| Niue | 14.56 (12.61, 16.59) | 22.15 (19.13, 25.2) | 34.27 | 0.89(0.86, 0.93) |
| North Korea | 11.73 (10.27, 13.24) | 14.79 (12.97, 16.66) | 20.71 | 0.99(0.94, 1.04) |
| North Macedonia | 11.76 (10.33, 13.35) | 21.15 (18.73, 23.94) | 44.39 | 1.46(1.39, 1.52) |
| Northern Mariana Islands | 19.32 (16.63, 22.17) | 24.42 (21.6, 27.51) | 20.88 | 2.04(2.01, 2.08) |
| Norway | 15.37 (13.67, 17.09) | 19.15 (17.32, 21.1) | 19.72 | 0.73(0.54, 0.93) |
| Oman | 18.74 (16.65, 21.12) | 37.27 (33.43, 41.27) | 49.73 | 0.76(0.73, 0.79) |
| Pakistan | 11.19 (9.96, 12.47) | 14.88 (13.28, 16.56) | 24.85 | 2.4(2.34, 2.46) |
| Palau | 16.61 (14.11, 19.22) | 23.57 (20.55, 26.73) | 29.52 | 1(0.97, 1.02) |
| Palestine | 24.09 (21.56, 26.93) | 38.32 (34.38, 42.62) | 37.14 | 1.22(1.12, 1.31) |
| Panama | 18.86 (16.5, 21.53) | 27.57 (24.74, 31.04) | 31.6 | 1.62(1.6, 1.64) |
| Papua New Guinea | 6.71 (5.82, 7.75) | 9.36 (8.15, 10.89) | 28.28 | 1.32(1.29, 1.35) |
| Paraguay | 14.74 (12.85, 16.85) | 23.69 (20.93, 26.58) | 37.79 | 1.16(1.06, 1.26) |
| Peru | 11.47 (10.2, 12.91) | 22.16 (19.73, 24.8) | 48.23 | 1.65(1.62, 1.68) |
| Philippines | 14.28 (13.06, 15.6) | 20.18 (18.4, 21.99) | 29.2 | 2.31(2.25, 2.36) |
| Poland | 12.42 (10.91, 14.11) | 17.21 (15.29, 19.3) | 27.86 | 1.19(1.17, 1.21) |
| Portugal | 18.32 (16.17, 20.59) | 21 (18.57, 23.58) | 12.78 | 1.13(1.07, 1.18) |
| Puerto Rico | 17.3 (15.13, 19.53) | 25.5 (22.43, 28.8) | 32.18 | 0.45(0.38, 0.52) |
| Qatar | 26.84 (23.84, 30.17) | 44.21 (39.78, 49.16) | 39.28 | 1.35(1.31, 1.39) |
| Republic of Congo | 7.91 (7.06, 8.83) | 11.18 (9.91, 12.5) | 29.21 | 1.74(1.68, 1.8) |
| Reunion | 12.03 (10.58, 13.62) | 19.35 (17, 21.94) | 37.84 | 0.49(0.41, 0.57) |
| Romania | 9.71 (8.5, 11.08) | 16.62 (14.71, 18.67) | 41.56 | 1.21(1.16, 1.27) |
| Russia | 9.52 (8.6, 10.57) | 13.96 (12.56, 15.54) | 31.81 | 1.89(1.83, 1.95) |
| Rwanda | 7.6 (6.77, 8.47) | 10.08 (8.94, 11.27) | 24.66 | 1.34(1.25, 1.43) |
| Saint Kitts | 19.6 (16.33, 23.58) | 28.06 (23.58, 33.4) | 30.14 | 0.98(0.92, 1.05) |
| Saint Lucia | 15.28 (13.6, 17.26) | 23.3 (20.68, 26.46) | 34.42 | 1.22(1.11, 1.32) |
| Saint Vincent | 14.25 (12.48, 16.15) | 21.02 (18.63, 23.86) | 32.19 | 1.46(1.43, 1.48) |
| Samoa | 14.54 (12.67, 16.56) | 20.45 (17.95, 22.83) | 28.86 | 1.34(1.32, 1.37) |
| San Marino | 16.8 (14.84, 18.93) | 18.78 (16.68, 20.96) | 10.56 | 1.18(1.12, 1.24) |
| Sao Tome and Principe | 12.55 (11.15, 14.09) | 17.93 (15.86, 20.03) | 30.01 | 0.39(0.37, 0.41) |
| Saudi Arabia | 24.97 (22.18, 28.01) | 45.66 (41.39, 50.22) | 45.31 | 1.24(1.2, 1.27) |
| Senegal | 10.64 (9.47, 11.82) | 13.42 (11.96, 14.92) | 20.7 | 2.1(2.08, 2.12) |
| Serbia | 11.47 (10.09, 12.9) | 19.85 (17.54, 22.27) | 42.22 | 0.8(0.77, 0.84) |
| Seychelles | 16.64 (14.62, 18.82) | 25.15 (22.34, 28) | 33.82 | 1.92(1.88, 1.95) |
| Sierra Leone | 9.67 (8.61, 10.88) | 12.84 (11.41, 14.49) | 24.65 | 1.43(1.4, 1.45) |
| Singapore | 21.79 (19.93, 23.79) | 25.54 (23.05, 28.26) | 14.68 | 0.98(0.94, 1.01) |
| Slovakia | 12.19 (10.73, 14.02) | 18.25 (15.99, 20.88) | 33.2 | 0.55(0.54, 0.56) |
| Slovenia | 11.32 (9.99, 12.78) | 18.06 (15.94, 20.15) | 37.31 | 1.4(1.38, 1.42) |
| Solomon Islands | 11.5 (9.96, 13.38) | 13.74 (11.94, 15.83) | 16.3 | 1.63(1.59, 1.67) |
| Somalia | 7.63 (6.83, 8.49) | 8.91 (7.96, 9.93) | 14.37 | 0.61(0.58, 0.65) |
| South Africa | 13.68 (12.35, 15.08) | 19.12 (17.42, 20.99) | 28.41 | 0.54(0.51, 0.56) |
| South Korea | 18.84 (16.63, 21.38) | 21.65 (19.62, 23.81) | 12.98 | 1.16(1.09, 1.23) |
| South Sudan | 8.06 (7.17, 9.01) | 9.56 (8.5, 10.69) | 15.67 | 0.59(0.55, 0.63) |
| Spain | 20.85 (18.82, 22.97) | 21.47 (19.17, 23.8) | 2.89 | 0.1(0.07, 0.12) |
| Sri Lanka | 14.84 (13.17, 16.52) | 22.51 (19.87, 25.07) | 34.08 | 1.42(1.36, 1.49) |
| Sudan | 17.16 (15.09, 19.31) | 31.53 (27.95, 35.42) | 45.59 | 2.13(2.1, 2.16) |
| Suriname | 12.02 (10.08, 14.6) | 22.82 (19.33, 27.21) | 47.31 | 2.22(2.18, 2.27) |
| Swaziland | 14.37 (12.7, 16.34) | 18.67 (16.48, 20.99) | 23.02 | 0.08(0.07, 0.09) |
| Sweden | 15.93 (14.1, 17.91) | 16.29 (14.4, 18.29) | 2.2 | 0.44(0.44, 0.45) |
| Switzerland | 20.36 (18.2, 22.78) | 23.17 (20.76, 25.74) | 12.09 | 1.73(1.69, 1.78) |
| Syria | 22.09 (19.66, 24.81) | 36.27 (32.86, 40.12) | 39.1 | 0.52(0.51, 0.53) |
| Taiwan | 22.16 (20.04, 24.34) | 25.75 (23.65, 28.07) | 13.95 | 1.91(1.77, 2.06) |
| Tajikistan | 5.91 (4.98, 7.04) | 10.15 (8.56, 11.86) | 41.76 | 1.47(1.39, 1.55) |
| Tanzania | 8.37 (7.46, 9.34) | 10.62 (9.43, 11.79) | 21.14 | 1.3(1.23, 1.37) |
| Thailand | 15.72 (13.89, 17.76) | 24.06 (21.56, 26.69) | 34.65 | 0.85(0.81, 0.89) |
| Timor-Leste | 10.83 (9.62, 12.18) | 15.81 (13.96, 17.89) | 31.5 | 1.66(1.63, 1.68) |
| Togo | 10.84 (9.58, 12.18) | 13.84 (12.23, 15.41) | 21.7 | 1.16(1.14, 1.18) |
| Tonga | 13.99 (12.29, 15.88) | 19.56 (17.35, 22.06) | 28.48 | 1.62(1.56, 1.67) |
| Trinidad | 13.8 (12.12, 15.6) | 22.05 (19.39, 24.97) | 37.41 | 1.97(1.93, 2.02) |
| Tunisia | 21.21 (18.78, 23.8) | 37.26 (33.3, 41.16) | 43.06 | 2.24(2.19, 2.28) |
| Turkey | 18.96 (17.14, 20.97) | 35.87 (32.17, 39.93) | 47.14 | 1.45(1.39, 1.52) |
| Turkmenistan | 8.02 (6.82, 9.39) | 12.13 (10.45, 14.1) | 33.94 | 1.65(1.62, 1.69) |
| Tuvalu | 11.43 (10.03, 13.05) | 18.45 (16.28, 20.99) | 38.04 | 0.81(0.77, 0.86) |
| Uganda | 7.19 (6.43, 7.98) | 9.09 (8.08, 10.16) | 20.88 | 0.78(0.74, 0.82) |
| UK | 18.35 (16.51, 20.29) | 19.96 (18.01, 22.07) | 8.08 | 1.48(1.42, 1.54) |
| Ukraine | 7.44 (6.41, 8.58) | 9.29 (8.13, 10.59) | 19.99 | 0.28(0.26, 0.31) |
| United Arab Emirates | 28.49 (25.36, 31.68) | 43.84 (39.65, 48.39) | 35.01 | 0.82(0.77, 0.87) |
| Uruguay | 17.2 (15.54, 19.19) | 20.88 (18.86, 23.22) | 17.64 | 0.25(0.18, 0.32) |
| USA | 25.85 (23.53, 28.36) | 27.85 (25.53, 30.31) | 7.2 | 1.5(1.48, 1.52) |
| Uzbekistan | 10.14 (8.74, 11.81) | 15.17 (13.13, 17.64) | 33.13 | 0.67(0.66, 0.69) |
| Vanuatu | 10.96 (9.62, 12.53) | 16.05 (14.07, 18.26) | 31.69 | 1.39(1.37, 1.41) |
| Venezuela | 18.83 (16.54, 21.48) | 28.56 (25.59, 31.94) | 34.09 | 1.34(1.26, 1.42) |
| Vietnam | 10.09 (8.97, 11.35) | 15.94 (14.16, 17.85) | 36.71 | 1.44(1.42, 1.47) |
| Virgin Islands | 14.81 (13.08, 16.8) | 22.83 (20.18, 25.92) | 35.16 | 1.59(1.53, 1.66) |
| Yemen | 16.72 (14.8, 18.87) | 28.89 (25.46, 32.55) | 42.13 | 1.91(1.88, 1.94) |
| Zambia | 8.76 (7.84, 9.8) | 11.39 (10.08, 12.79) | 23.07 | 0.92(0.88, 0.95) |
| Zimbabwe | 13.1 (11.58, 14.78) | 15.41 (13.68, 17.32) | 14.96 | 0.55(0.48, 0.63) |

AAPC, average annual percentage changes; #, Change percentage was calculated by 100$\times$(ASDR2019-ASDR1990) / ASDR2019; *, the AAPC values and their 95% CI were more than 0 were defined as “significant increase” group. The AAPC values and their 95% CI were less than 0 was defined as “significant decreasing group”.

**Supplementary table 2.** Trend of age-standardized deaths rate (ASDR) from 1990 to 2019 in the 204 countries.

| location | ASDR 1990  ($\times100, 000$) | ASDR 2019  ($\times100, 000$) | Change percentage  (%)# | AAPCs  (%)* |
| --- | --- | --- | --- | --- |
| Afghanistan | 18.27 (12.63, 28.81) | 16.39 (11.54, 26.05) | -11.5 | -0.36(-0.45, -0.27) |
| Albania | 3.01 (2.36, 3.76) | 2.18 (1.51, 3.03) | -38.1 | -1.23(-1.82, -0.63) |
| Algeria | 14.07 (10.19, 19.77) | 11.63 (8.52, 15.44) | -20.98 | -0.64(-0.76, -0.53) |
| American Samoa | 14.77 (11.56, 18.11) | 21.38 (17.14, 26.06) | 30.89 | 1.33(1.05, 1.61) |
| Andorra | 3.88 (2.68, 5.25) | 3.48 (2.49, 4.65) | -11.24 | -0.36(-0.46, -0.26) |
| Angola | 9.9 (7.05, 12.72) | 9.27 (6.2, 12.16) | -6.86 | -0.22(-0.37, -0.08) |
| Antigua | 6.92 (5.57, 8.38) | 11.03 (8.61, 13.61) | 37.25 | 1.77(0.75, 2.79) |
| Argentina | 8.06 (6.58, 9.66) | 10.32 (8.33, 12.37) | 21.95 | 0.88(0.56, 1.19) |
| Armenia | 0.48 (0.35, 0.65) | 1.59 (1.12, 2.19) | 69.89 | 4.22(3.83, 4.62) |
| Australia | 2.11 (1.67, 2.61) | 2.95 (2.07, 4.03) | 28.66 | 1.31(0.96, 1.65) |
| Austria | 2.06 (1.6, 2.53) | 5.41 (4.06, 6.86) | 61.89 | 3.3(3.03, 3.57) |
| Azerbaijan | 0.97 (0.71, 1.33) | 2.27 (1.59, 3.2) | 57.28 | 3.02(2.32, 3.72) |
| Bahamas | 6.12 (4.93, 7.45) | 7.86 (6.08, 9.96) | 22.15 | 0.87(0.33, 1.41) |
| Bahrain | 13.46 (10.37, 17.22) | 12.58 (9.56, 15.96) | -6.99 | -0.18(-0.92, 0.57) |
| Bangladesh | 3.32 (2.31, 4.39) | 2.84 (2.09, 3.77) | -16.97 | -0.36(-1, 0.28) |
| Barbados | 4.89 (3.99, 5.91) | 7.03 (5.44, 8.67) | 30.4 | 1.24(0.73, 1.75) |
| Belarus | 0.67 (0.51, 0.84) | 0.85 (0.63, 1.09) | 21.08 | 0.73(0.03, 1.43) |
| Belgium | 3.09 (2.43, 3.83) | 3.76 (2.86, 4.75) | 17.7 | 0.65(0.15, 1.14) |
| Belize | 6.01 (4.81, 7.34) | 10.27 (8.06, 12.55) | 41.47 | 1.99(1.59, 2.39) |
| Benin | 14.68 (11.88, 17.77) | 13.43 (10.2, 17.17) | -9.34 | -0.3(-0.42, -0.19) |
| Bermuda | 4.75 (3.87, 5.72) | 4.31 (3.29, 5.45) | -10.08 | -0.32(-0.72, 0.08) |
| Bhutan | 5.67 (3.61, 8.33) | 7.2 (4.96, 9.81) | 21.2 | 0.82(0.71, 0.92) |
| Bolivia | 10.42 (7.94, 13.5) | 16.24 (11.88, 21.57) | 35.85 | 1.54(1.43, 1.65) |
| Bosnia and Herzegovina | 2.14 (1.65, 2.7) | 2.82 (2, 3.89) | 24.23 | 0.86(0.23, 1.49) |
| Botswana | 11.82 (8.44, 16.74) | 15.21 (10.44, 20.77) | 22.31 | 0.9(0.7, 1.09) |
| Brazil | 5.08 (4.23, 5.95) | 5.23 (4.28, 6.25) | 2.82 | 0.14(-0.14, 0.41) |
| Brunei | 10.21 (7.89, 12.9) | 10.86 (8.34, 13.74) | 5.99 | 0.24(-0.08, 0.55) |
| Bulgaria | 1.69 (1.32, 2.1) | 3.58 (2.71, 4.69) | 52.81 | 2.77(2.34, 3.21) |
| Burkina Faso | 12.64 (10.14, 15.49) | 12.79 (10.21, 15.77) | 1.14 | 0.09(-0.06, 0.23) |
| Burundi | 10.51 (7.69, 13.91) | 9.05 (6.72, 11.88) | -16.24 | -0.52(-0.64, -0.41) |
| Cambodia | 8.51 (6.76, 10.49) | 7.79 (6.08, 9.46) | -9.26 | 1.83(1.21, 2.44) |
| Cameroon | 19.43 (14.7, 24.54) | 17.13 (12.68, 22.36) | -13.39 | -0.3(-0.4, -0.21) |
| Canada | 3.65 (3, 4.3) | 4.41 (3.52, 5.25) | 17.19 | -0.44(-0.62, -0.26) |
| Cape Verde | 6.37 (5.29, 7.89) | 10.45 (8.67, 12.43) | 39 | 0.66(0.51, 0.81) |
| Central African Republic | 11.13 (8.28, 14.33) | 11.12 (7.84, 14.85) | -0.16 | -0.02(-0.09, 0.05) |
| Chad | 13.32 (9.59, 19.08) | 13.01 (9.64, 17.22) | -2.4 | -0.06(-0.15, 0.04) |
| Chile | 5.54 (4.54, 6.61) | 8.22 (6.64, 9.88) | 32.66 | 1.37(1.05, 1.69) |
| China | 4.29 (3.55, 5.07) | 4.09 (3.32, 4.88) | -4.99 | -0.16(-0.34, 0.02) |
| Colombia | 6.1 (4.95, 7.39) | 5.22 (3.75, 7.03) | -16.86 | -0.59(-0.99, -0.18) |
| Comoros | 10.23 (7.07, 13.79) | 9.53 (7.45, 11.98) | -7.27 | -0.24(-0.5, 0.03) |
| Cook Islands | 8.37 (6.67, 10.26) | 10.73 (8.51, 13.25) | 22.01 | -0.36(-0.58, -0.13) |
| Costa Rica | 4.37 (3.54, 5.36) | 7.75 (5.64, 10.39) | 43.65 | 0.87(0.69, 1.05) |
| Croatia | 2 (1.56, 2.51) | 3 (2.19, 3.96) | 33.11 | 2.13(1.81, 2.46) |
| Cuba | 2 (1.66, 2.39) | 4 (3.03, 5.14) | 49.87 | -0.54(-0.76, -0.33) |
| Cyprus | 11 (8.08, 14.56) | 7.04 (5.28, 9.1) | -56.18 | 1.62(0.64, 2.6) |
| Czech Republic | 2.07 (1.61, 2.58) | 1.78 (1.32, 2.36) | -16.3 | 2.58(2.26, 2.91) |
| Democratic Republic of the Congo | 11.12 (8.9, 13.6) | 9.18 (6.78, 12.02) | -21.06 | -1.65(-1.92, -1.38) |
| Denmark | 1.26 (0.97, 1.6) | 2.85 (2.15, 3.58) | 55.72 | -0.51(-0.83, -0.19) |
| Djibouti | 8.48 (5.99, 11.51) | 10.2 (7.51, 13.61) | 16.91 | -0.24(-0.3, -0.17) |
| Dominica | 9.37 (7.5, 11.4) | 12.81 (9.83, 16.22) | 26.84 | -0.66(-0.77, -0.54) |
| Dominican Republic | 4 (3.2, 4.97) | 6.78 (4.89, 9.26) | 40.99 | 2.86(2.34, 3.39) |
| Ecuador | 5.86 (4.82, 7.03) | 15.4 (11.71, 19.74) | 61.94 | 0.64(0.55, 0.74) |
| Egypt | 13.88 (9.24, 18.28) | 15.47 (9.38, 22.05) | 10.28 | 1.07(0.86, 1.28) |
| El Salvador | 4.97 (4.02, 6.07) | 16.81 (11.98, 23.2) | 70.44 | 1.87(1.46, 2.28) |
| Equatorial Guinea | 10.36 (7.43, 13.54) | 12.29 (8.62, 16.69) | 15.71 | 3.33(2.88, 3.78) |
| Eritrea | 8.26 (4.96, 12.55) | 9.73 (6.65, 13.57) | 15.11 | 0.4(-0.19, 1) |
| Estonia | 0.82 (0.65, 1.03) | 3.63 (2.72, 4.78) | 77.34 | 4.48(3.57, 5.4) |
| Ethiopia | 13.34 (10.42, 16.55) | 9.6 (7.74, 11.7) | -38.88 | 0.64(0.54, 0.73) |
| Fiji | 11.68 (9.05, 15.02) | 16.3 (12.54, 20.6) | 28.37 | 0.55(0.43, 0.67) |
| Finland | 0.74 (0.56, 0.97) | 1.32 (0.93, 1.76) | 43.49 | 5.34(4.27, 6.42) |
| France | 3.08 (2.4, 3.76) | 2.62 (2.01, 3.28) | -17.51 | 0.9(0.71, 1.08) |
| Gabon | 12.64 (8.63, 16.39) | 15.27 (9.1, 20.57) | 17.2 | -1.14(-1.36, -0.92) |
| Gambia | 13.88 (10.69, 17.57) | 14.44 (11.53, 18.26) | 3.87 | 1.15(0.85, 1.45) |
| Georgia | 0.73 (0.55, 0.97) | 1.42 (1.01, 1.91) | 48.64 | 2.04(1.61, 2.48) |
| Germany | 1.85 (1.42, 2.36) | 4.65 (3.39, 6.05) | 60.3 | -0.57(-0.89, -0.25) |
| Ghana | 11.02 (8.15, 14.73) | 12.18 (8.54, 15.65) | 9.58 | 0.65(0.48, 0.83) |
| Greece | 6.39 (4.77, 8.05) | 4.73 (3.58, 6.05) | -35.08 | 0.17(-0.27, 0.62) |
| Greenland | 5.3 (4.19, 6.45) | 6.09 (4.74, 7.49) | 12.97 | 2.1(0.76, 3.46) |
| Grenada | 8.84 (7.08, 10.79) | 12.34 (9.95, 15.06) | 28.42 | 3.34(2.92, 3.76) |
| Guam | 9.48 (7.82, 11.21) | 10.56 (8.29, 13.18) | 10.25 | 0.34(0.16, 0.51) |
| Guatemala | 8.81 (7.16, 10.89) | 15.22 (11.37, 19.89) | 42.08 | -1.17(-1.88, -0.45) |
| Guinea | 15.45 (11.73, 20.61) | 13.9 (10.52, 17.55) | -11.16 | 0.54(0.31, 0.76) |
| Guinea-Bissau | 20.04 (15.59, 25.3) | 16.1 (12.32, 20.25) | -24.41 | 1.21(0.6, 1.82) |
| Guyana | 7.33 (5.75, 9.01) | 11.61 (8.67, 15.05) | 36.85 | 0.34(-0.2, 0.9) |
| Haiti | 8.13 (5.46, 13.14) | 8.72 (5.77, 13.88) | 6.7 | 1.85(1.39, 2.31) |
| Honduras | 6.75 (4.71, 10.94) | 15.02 (11.13, 20.4) | 55.06 | -0.35(-0.46, -0.24) |
| Hungary | 1.54 (1.23, 1.89) | 2.76 (2.06, 3.56) | 44.13 | -0.75(-0.81, -0.7) |
| Iceland | 1.63 (1.29, 2) | 2.06 (1.54, 2.57) | 20.61 | 1.68(0.98, 2.38) |
| India | 6.06 (4.73, 7.61) | 5.2 (4.02, 6.51) | -16.57 | 0.25(0.2, 0.3) |
| Indonesia | 7.69 (6.39, 9.16) | 8 (6.56, 9.47) | 3.86 | 2.85(2.49, 3.21) |
| Iran | 8.23 (6.79, 10.3) | 7.08 (5.85, 8.25) | -16.32 | 2.1(1.68, 2.52) |
| Iraq | 15.2 (11.31, 21.54) | 14.35 (10.63, 19.39) | -5.88 | 0.82(0.51, 1.14) |
| Ireland | 3.61 (2.76, 4.55) | 3.11 (2.33, 4.03) | -16.05 | -0.5(-1.57, 0.58) |
| Israel | 8.09 (6.32, 9.82) | 7.85 (5.98, 9.74) | -3.07 | 0.17(-0.04, 0.38) |
| Italy | 3.37 (2.74, 4) | 3.23 (2.54, 3.91) | -4.07 | -0.49(-0.75, -0.22) |
| Ivory Coast | 17.25 (13.7, 21.27) | 14.64 (11.47, 18.27) | -17.77 | -0.2(-0.33, -0.07) |
| Jamaica | 6.56 (5.33, 7.84) | 7.63 (5.76, 9.82) | 14.01 | -0.5(-0.92, -0.08) |
| Japan | 4.2 (3.34, 5.05) | 3.04 (2.24, 3.75) | -38.12 | -0.32(-0.73, 0.08) |
| Jordan | 13.89 (10.7, 17.41) | 13.01 (10.16, 16.07) | -6.79 | -0.15(-0.44, 0.14) |
| Kazakhstan | 0.74 (0.57, 0.96) | 1.51 (1.1, 2.04) | 50.77 | 0.78(-0.43, 2.02) |
| Kenya | 7.15 (5.56, 9.12) | 8.67 (6.9, 10.71) | 17.54 | -1.12(-1.35, -0.9) |
| Kiribati | 14 (10.71, 17.56) | 18.66 (13.23, 24.49) | 24.95 | -0.17(-0.7, 0.37) |
| Kuwait | 9.67 (7.64, 11.7) | 6.01 (4.63, 7.65) | -60.93 | 2.5(1.57, 3.43) |
| Kyrgyzstan | 1.04 (0.79, 1.37) | 1.04 (0.77, 1.41) | 0.41 | 0.67(0.57, 0.77) |
| Laos | 19.25 (14.21, 26.13) | 16.29 (12.6, 20.87) | -18.16 | 0.98(0.88, 1.09) |
| Latvia | 0.58 (0.43, 0.75) | 1.41 (1.01, 1.88) | 58.92 | -1.58(-2.5, -0.66) |
| Lebanon | 11.43 (8.82, 14.59) | 8.51 (6, 11.4) | -34.29 | 0.17(-0.56, 0.91) |
| Lesotho | 10.62 (8.36, 13.6) | 19.9 (14.43, 25.86) | 46.62 | -0.57(-0.62, -0.52) |
| Liberia | 17.2 (13.65, 21.72) | 13.89 (10.17, 19.14) | -23.78 | 2.93(2.31, 3.55) |
| Libya | 10.35 (7.44, 14.41) | 10.56 (6.92, 14.89) | 1.95 | -1.01(-1.1, -0.92) |
| Lithuania | 0.75 (0.58, 0.94) | 1.14 (0.87, 1.5) | 33.77 | 2.18(2.05, 2.31) |
| Luxembourg | 3.3 (2.47, 4.2) | 3.44 (2.51, 4.42) | 3.86 | -0.78(-0.94, -0.61) |
| Madagascar | 7.96 (5.73, 10.6) | 7.34 (5.29, 9.84) | -8.45 | 0.11(-0.36, 0.57) |
| Malawi | 9.59 (7.57, 11.81) | 9.38 (7.28, 11.65) | -2.23 | 1.53(0.81, 2.26) |
| Malaysia | 9.82 (8.15, 11.46) | 10.38 (8.1, 13.1) | 5.42 | 0.17(-0.04, 0.37) |
| Maldives | 23.23 (18.81, 29.04) | 14.47 (11.55, 17.72) | -60.6 | -0.23(-0.42, -0.04) |
| Mali | 15.2 (11.77, 20.09) | 12.87 (10.01, 16.31) | -18.11 | -0.08(-0.29, 0.13) |
| Malta | 4.29 (3.23, 5.46) | 3.35 (2.49, 4.34) | -28.34 | 0.24(-0.81, 1.3) |
| Marshall Islands | 13.64 (10.12, 18.68) | 18.33 (13.12, 25.41) | 25.56 | -1.69(-1.96, -1.42) |
| Mauritania | 19.66 (15.58, 23.56) | 13.24 (9.93, 16.91) | -48.43 | -0.56(-0.7, -0.41) |
| Mauritius | 17.36 (14.7, 20.21) | 28.1 (21.91, 35.16) | 38.23 | -0.86(-1.05, -0.68) |
| Mexico | 8.31 (6.96, 9.7) | 16.31 (12.98, 20.17) | 49.04 | 0.93(0.74, 1.12) |
| Micronesia | 16.74 (12.77, 22.52) | 26.31 (18.93, 34.3) | 36.38 | -1.41(-1.55, -1.26) |
| Moldova | 0.77 (0.6, 0.97) | 1.04 (0.8, 1.31) | 25.99 | 1.58(0.99, 2.18) |
| Monaco | 1.35 (0.98, 1.79) | 2.14 (1.6, 2.77) | 36.65 | 2.37(2.12, 2.63) |
| Mongolia | 4.43 (3.26, 5.94) | 2.35 (1.64, 3.23) | -88.88 | 1.57(1.53, 1.61) |
| Montenegro | 3.53 (2.75, 4.41) | 4.11 (3.09, 5.3) | 14.15 | 1.6(1.49, 1.71) |
| Morocco | 9.87 (7.37, 14.65) | 12.27 (9.2, 16.5) | 19.61 | -2.2(-2.83, -1.56) |
| Mozambique | 8.13 (6.24, 10.7) | 9.18 (6.93, 11.89) | 11.49 | 0.74(0.22, 1.27) |
| Myanmar | 11.24 (8.35, 14.61) | 10.25 (8.34, 12.59) | -9.69 | 0.72(0.16, 1.27) |
| Namibia | 12.38 (8.95, 17.39) | 12.15 (8.56, 17.25) | -1.82 | 0.47(0.36, 0.57) |
| Nauru | 16.63 (12.52, 21.3) | 22.78 (16.21, 29.04) | 26.96 | -0.33(-0.44, -0.22) |
| Nepal | 4.62 (3.18, 6.67) | 6.51 (4.44, 8.95) | 29.09 | -0.03(-0.23, 0.17) |
| Netherlands | 3.23 (2.52, 4.01) | 3.9 (2.99, 4.85) | 17.26 | 1.09(0.96, 1.23) |
| New Zealand | 2.14 (1.66, 2.67) | 2.96 (2.25, 3.69) | 27.77 | 1.2(1.12, 1.29) |
| Nicaragua | 9.74 (7.76, 12.04) | 22.32 (17.04, 28.42) | 56.37 | 0.75(0.45, 1.06) |
| Niger | 14.49 (10.95, 19.22) | 12.08 (9.1, 15.67) | -19.97 | 1.09(0.55, 1.64) |
| Nigeria | 11.4 (8.99, 14.63) | 9.83 (7.66, 12.28) | -16.04 | 2.86(2.02, 3.71) |
| Niue | 12.37 (9.23, 15.76) | 16.66 (11.88, 21.73) | 25.73 | -0.59(-0.7, -0.48) |
| North Korea | 5.42 (4.19, 6.91) | 5.05 (3.89, 6.4) | -7.15 | -0.5(-0.62, -0.39) |
| North Macedonia | 3.45 (2.75, 4.28) | 3.98 (2.97, 5.2) | 13.32 | 1.03(0.91, 1.15) |
| Northern Mariana Islands | 16.53 (13.22, 20.45) | 20.96 (16.71, 25.17) | 21.13 | 0.4(0.11, 0.7) |
| Norway | 1.58 (1.3, 1.89) | 2.48 (1.97, 3) | 36.13 | 0.82(0.77, 0.87) |
| Oman | 5.93 (4.3, 7.99) | 7.17 (5.58, 8.76) | 17.35 | 1.62(1.32, 1.92) |
| Pakistan | 6.85 (4.76, 9.97) | 9.91 (7.07, 13.05) | 30.86 | 0.59(0.2, 0.98) |
| Palau | 18.83 (14.5, 24.14) | 24.04 (18.56, 30.29) | 21.67 | 1.3(1.2, 1.4) |
| Palestine | 16.4 (12.7, 20.78) | 11.74 (9.16, 14.47) | -39.68 | 0.85(0.74, 0.96) |
| Panama | 4.02 (3.24, 4.97) | 7.6 (5.58, 10.19) | 47.07 | -1.15(-1.37, -0.93) |
| Papua New Guinea | 3.87 (2.97, 5.05) | 4.65 (3.43, 6.12) | 16.71 | 2.38(2.17, 2.6) |
| Paraguay | 4.27 (3.37, 5.23) | 9.4 (6.87, 12.39) | 54.59 | 0.62(0.55, 0.7) |
| Peru | 6.84 (5.42, 8.6) | 7.53 (5.38, 10.12) | 9.1 | 2.94(2.2, 3.67) |
| Philippines | 15.2 (12.81, 17.79) | 17.28 (13.87, 21.57) | 12.02 | 0.27(-0.54, 1.09) |
| Poland | 2.72 (2.24, 3.22) | 1.92 (1.47, 2.43) | -42.19 | 0.55(0.05, 1.05) |
| Portugal | 3.85 (2.96, 4.82) | 4.62 (3.46, 5.88) | 16.64 | -1.18(-1.54, -0.82) |
| Puerto Rico | 8.05 (6.55, 9.52) | 7.53 (5.44, 9.82) | -6.8 | 0.62(0.41, 0.83) |
| Qatar | 17.06 (12.34, 30.05) | 17.51 (13.24, 22.45) | 2.56 | -0.23(-0.51, 0.06) |
| Republic of Congo | 13.17 (8.86, 17.06) | 11.9 (7.99, 15.93) | -10.68 | 0.35(0.07, 0.63) |
| Reunion | 11.22 (8.37, 14.95) | 14.15 (10.71, 18.92) | 20.66 | -0.26(-0.52, 0) |
| Romania | 1.7 (1.45, 1.98) | 1.98 (1.41, 2.64) | 13.85 | 0.99(-0.36, 2.37) |
| Russia | 1.05 (0.86, 1.26) | 1.23 (0.95, 1.53) | 14.68 | 0.41(-0.45, 1.27) |
| Rwanda | 10.93 (8.48, 13.79) | 8.92 (6.71, 11.34) | -22.45 | 0.67(0, 1.33) |
| Saint Kitts | 12.75 (10.27, 15.28) | 14.39 (11.36, 18.01) | 11.38 | -0.7(-0.94, -0.47) |
| Saint Lucia | 8.32 (6.7, 10.06) | 9.31 (7.15, 11.7) | 10.62 | 0.49(0.2, 0.78) |
| Saint Vincent | 6.58 (5.33, 7.91) | 9.5 (7.58, 11.55) | 30.73 | 0.48(0.08, 0.87) |
| Samoa | 13.27 (10.07, 17.5) | 15.66 (12.14, 20.17) | 15.28 | 1.26(0.84, 1.68) |
| San Marino | 1.36 (0.98, 1.79) | 1.75 (1.14, 2.53) | 22.37 | 0.56(0.47, 0.65) |
| Sao Tome and Principe | 18.87 (15.26, 22.36) | 22.5 (17.45, 27.36) | 16.15 | 0.89(0.73, 1.05) |
| Saudi Arabia | 16.88 (12.69, 21.86) | 19.29 (14.72, 24.09) | 12.52 | 0.64(0.36, 0.92) |
| Senegal | 17.41 (13.44, 23.2) | 14.56 (11.32, 18.84) | -19.54 | 0.48(0.25, 0.7) |
| Serbia | 3.95 (3.03, 5.12) | 5.15 (3.74, 6.94) | 23.28 | -0.64(-0.94, -0.34) |
| Seychelles | 12.22 (10.25, 14.44) | 18.2 (15, 21.7) | 32.86 | 1.16(0.64, 1.68) |
| Sierra Leone | 13.87 (10.86, 17.28) | 11.88 (9.24, 15.22) | -16.74 | 1.41(1.17, 1.65) |
| Singapore | 4.46 (3.55, 5.49) | 3.44 (2.64, 4.28) | -29.67 | -0.53(-0.63, -0.43) |
| Slovakia | 3.01 (2.33, 3.79) | 2.43 (1.74, 3.26) | -24.05 | -0.95(-1.58, -0.32) |
| Slovenia | 0.96 (0.72, 1.25) | 1.46 (1.02, 2) | 34.17 | -0.87(-1.41, -0.32) |
| Solomon Islands | 12.83 (9.39, 18.26) | 7.69 (6.13, 9.57) | -66.94 | 1.45(0.58, 2.32) |
| Somalia | 10.62 (7.49, 14.04) | 10.21 (7.42, 14) | -4.06 | -1.98(-3.02, -0.93) |
| South Africa | 7.85 (6.63, 9.22) | 11.65 (9.79, 13.67) | 32.6 | -0.14(-0.23, -0.05) |
| South Korea | 3.47 (2.88, 4.15) | 3.16 (2.54, 3.86) | -9.87 | 1.32(0.7, 1.95) |
| South Sudan | 9.76 (6.69, 13.39) | 9.86 (6.94, 13.59) | 1.02 | 0.01(-0.06, 0.09) |
| Spain | 4.43 (3.39, 5.59) | 3.74 (2.72, 4.92) | -18.37 | -0.62(-0.92, -0.31) |
| Sri Lanka | 10.26 (8.47, 12.18) | 9.59 (6.93, 12.81) | -7.05 | -0.31(-0.65, 0.02) |
| Sudan | 10.46 (7.58, 15.93) | 10.6 (7.11, 17.32) | 1.28 | -0.04(-0.27, 0.19) |
| Suriname | 7.25 (5.88, 8.81) | 11.68 (8.94, 14.77) | 37.96 | 1.68(1.15, 2.21) |
| Swaziland | 15.3 (11.67, 19.24) | 19.96 (14.42, 26.23) | 23.37 | 2.54(2.1, 2.97) |
| Sweden | 1.12 (0.9, 1.37) | 2.37 (1.82, 2.92) | 52.65 | 2.5(2.03, 2.98) |
| Switzerland | 1.8 (1.37, 2.26) | 3.57 (2.61, 4.6) | 49.57 | -0.81(-1.05, -0.57) |
| Syria | 12.83 (9.82, 17.19) | 10.27 (7.61, 13.26) | -24.94 | -0.65(-0.96, -0.33) |
| Taiwan | 9.66 (8.25, 11.26) | 7.98 (6.11, 10.43) | -21.06 | 4.54(3.86, 5.22) |
| Tajikistan | 0.37 (0.27, 0.49) | 1.3 (0.91, 1.81) | 71.63 | 0.08(-0.04, 0.19) |
| Tanzania | 8.59 (6.62, 11.01) | 6.57 (5.4, 7.96) | -30.79 | 0.06(-0.06, 0.18) |
| Thailand | 11.42 (9.36, 13.9) | 11.78 (8.63, 15.58) | 3.1 | -0.48(-0.65, -0.31) |
| Timor-Leste | 12.53 (9.27, 17.59) | 12.72 (9.56, 16.54) | 1.56 | 0.81(0.74, 0.88) |
| Togo | 14.52 (11.34, 18.84) | 12.68 (9.93, 15.89) | -14.52 | 1.37(1.04, 1.7) |
| Tonga | 10 (7.79, 13.23) | 14.65 (11.18, 19.66) | 31.72 | 1.58(0.95, 2.22) |
| Trinidad | 5.93 (4.87, 7.06) | 9.14 (6.49, 12.3) | 35.16 | -0.08(-0.3, 0.14) |
| Tunisia | 8.5 (6.52, 10.85) | 8.39 (5.85, 11.58) | -1.31 | -1.19(-1.54, -0.83) |
| Turkey | 11.72 (8.48, 17.9) | 8.24 (6.1, 10.63) | -42.11 | 0.97(0.31, 1.64) |
| Turkmenistan | 1.28 (0.97, 1.67) | 1.71 (1.22, 2.36) | 24.95 | 0.94(0.89, 0.99) |
| Tuvalu | 12.86 (9.53, 16.96) | 16.84 (11.93, 23.33) | 23.64 | 0.09(0, 0.18) |
| Uganda | 8.92 (6.48, 11.73) | 9.17 (6.9, 12.11) | 2.76 | 1.15(0.65, 1.65) |
| UK | 1.61 (1.27, 1.98) | 1.72 (1.32, 2.12) | 5.96 | -0.78(-1.69, 0.14) |
| Ukraine | 0.64 (0.52, 0.78) | 0.93 (0.73, 1.14) | 31.02 | 0.25(-0.5, 1) |
| United Arab Emirates | 21.12 (12.12, 27.41) | 16.98 (9.74, 26.63) | -24.35 | -0.9(-1.03, -0.77) |
| Uruguay | 4.85 (4.31, 5.35) | 6.21 (5.17, 7.41) | 21.88 | 1.76(1.5, 2.02) |
| USA | 4.11 (3.43, 4.72) | 6.87 (5.59, 8.03) | 40.22 | 1.41(1.15, 1.66) |
| Uzbekistan | 1.45 (1.01, 2.32) | 2.24 (1.62, 3.03) | 35.42 | 0.91(0.22, 1.62) |
| Vanuatu | 9.37 (6.52, 13.19) | 15.23 (11.05, 21.37) | 38.52 | 1.61(0.98, 2.23) |
| Venezuela | 4.29 (3.49, 5.23) | 9.69 (7.01, 13.2) | 55.75 | 1.69(1.5, 1.88) |
| Vietnam | 12.1 (9.42, 15.47) | 11.16 (8.48, 13.75) | -8.4 | 2.87(2.38, 3.37) |
| Virgin Islands | 5.63 (4.33, 7.13) | 8.29 (6.55, 10.24) | 32.09 | -0.28(-0.33, -0.22) |
| Yemen | 8.69 (6.11, 12.41) | 8.39 (6.1, 11.69) | -3.51 | -0.12(-0.18, -0.06) |
| Zambia | 11.35 (8.8, 14.31) | 10.77 (8.22, 13.83) | -5.45 | -0.2(-0.35, -0.06) |
| Zimbabwe | 12.04 (8.85, 18.18) | 16.01 (11.37, 23.88) | 24.81 | 1.01(0.77, 1.25) |

AAPC, average annual percentage changes; #, Change percentage was calculated by 100$\times$(ASDR2019-ASDR1990) / ASDR2019; *, the AAPC values and their 95% CI were more than 0 were defined as “significant increase” group. The AAPC values and their 95% CI were less than 0 was defined as “significant decreasing group”.


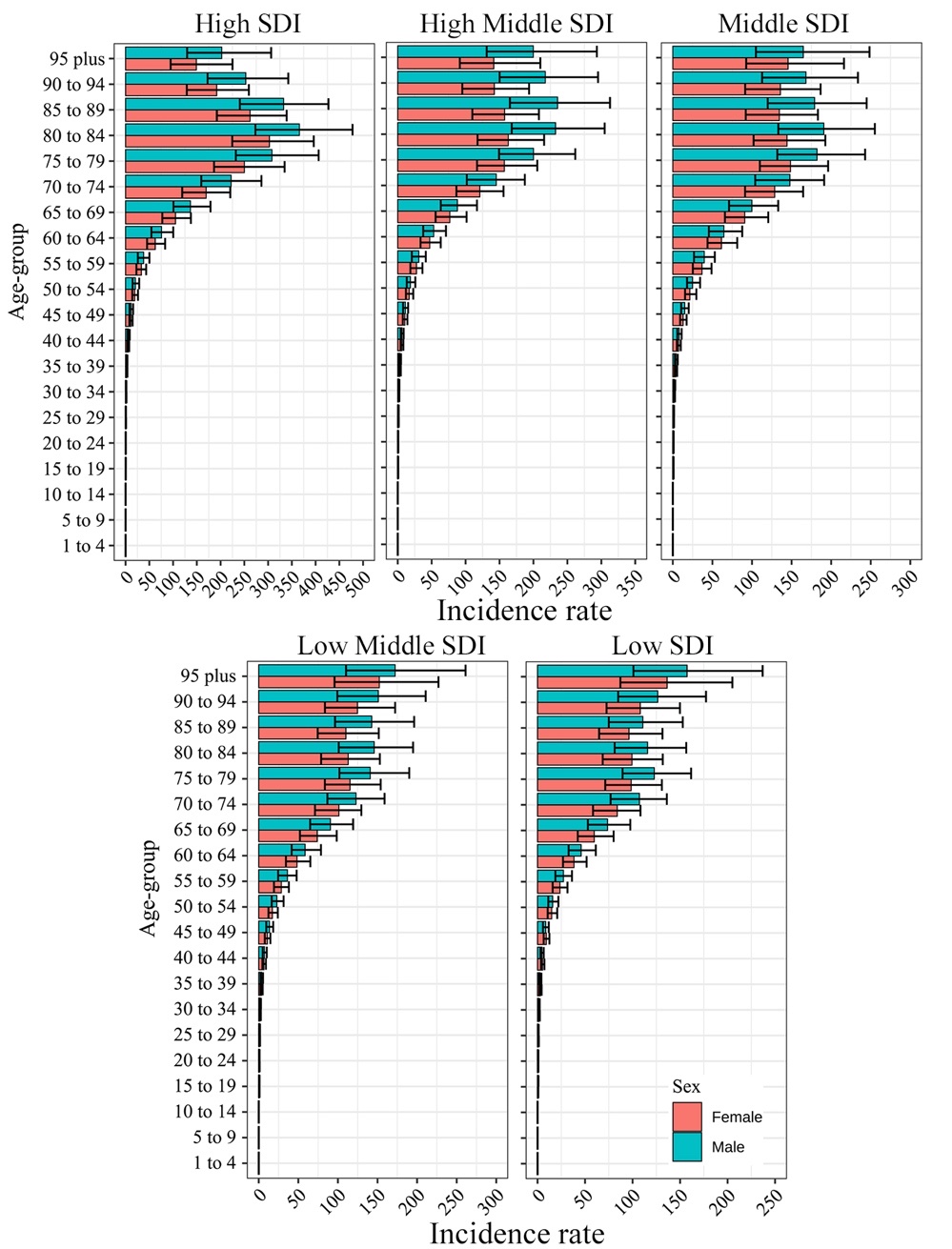


**Supplementary Figure 1.** The nonage-standardized incidence rate of CKDs due to hypertension in different age groups by 5 SDI regions in 2019.


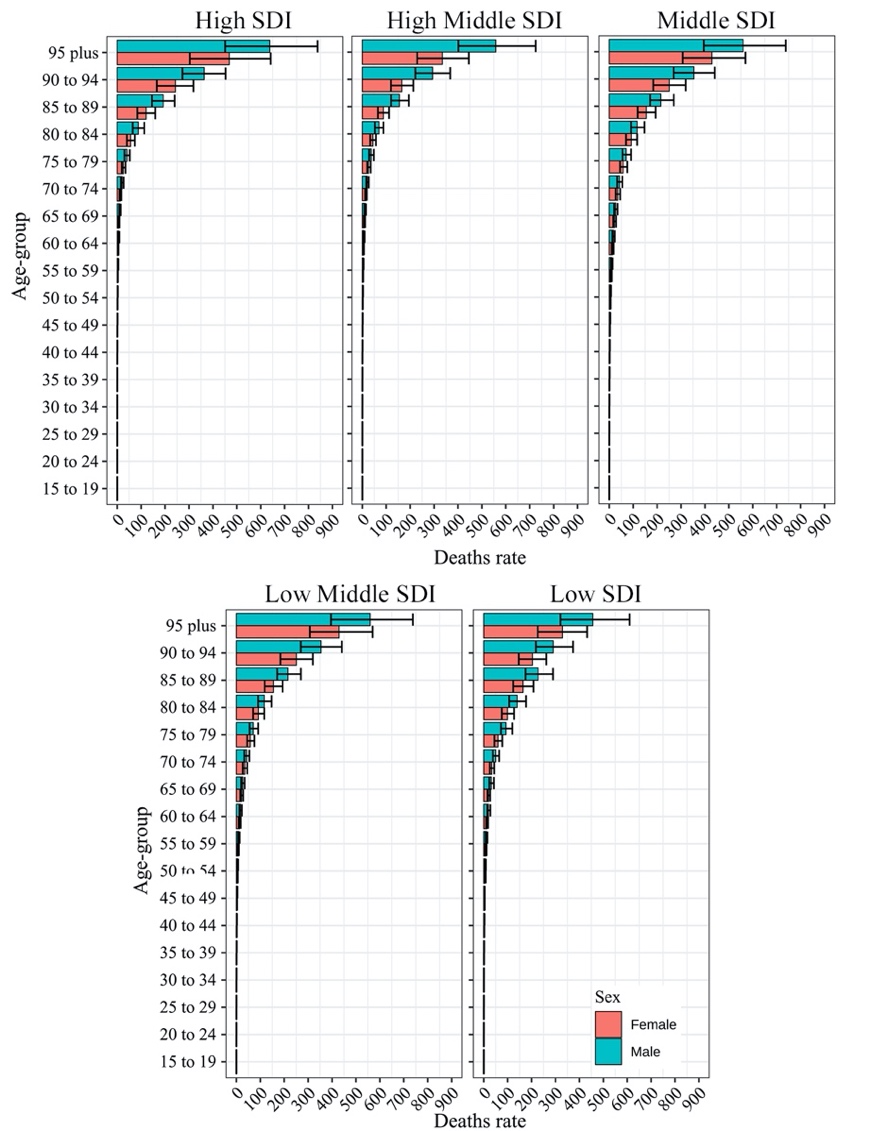


**Supplementary Figure 2.** The nonage-standardized deaths rate of CKDs due to hypertension in different age groups by 5 SDI regions in 2019.

**
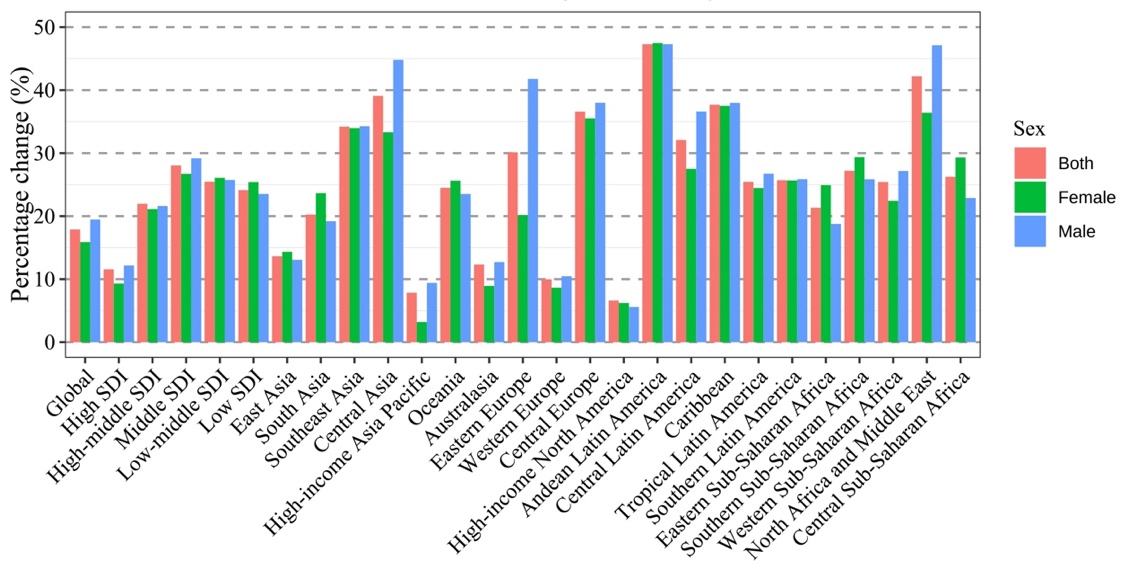
**

**Supplementary Figure 3.** The change percentage of age-standardized incidence rate of CKDs due to hypertension in 2019, which compared to 1990, by SDI and WHO geographic regions.


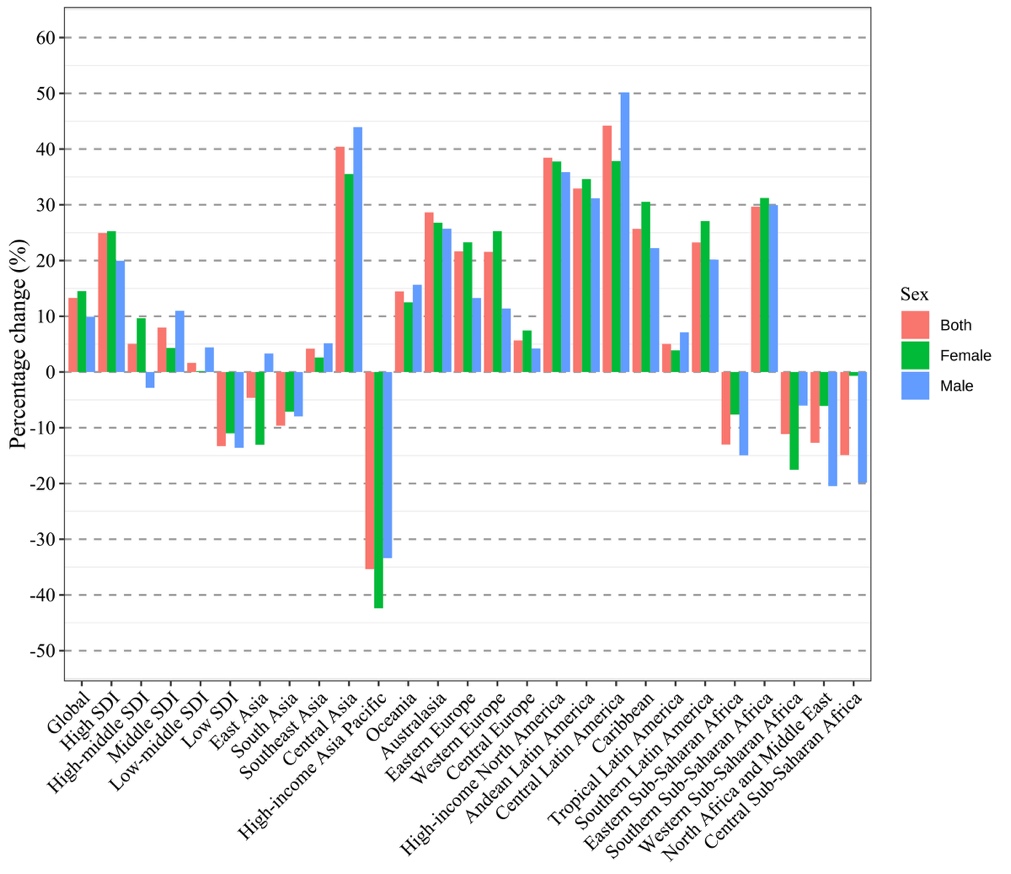


**Supplementary Figure 4.** The change percentage of age-standardized deaths rate of CKDs due to hypertension in 2019, which compared to 1990, by SDI and WHO geographic regions.


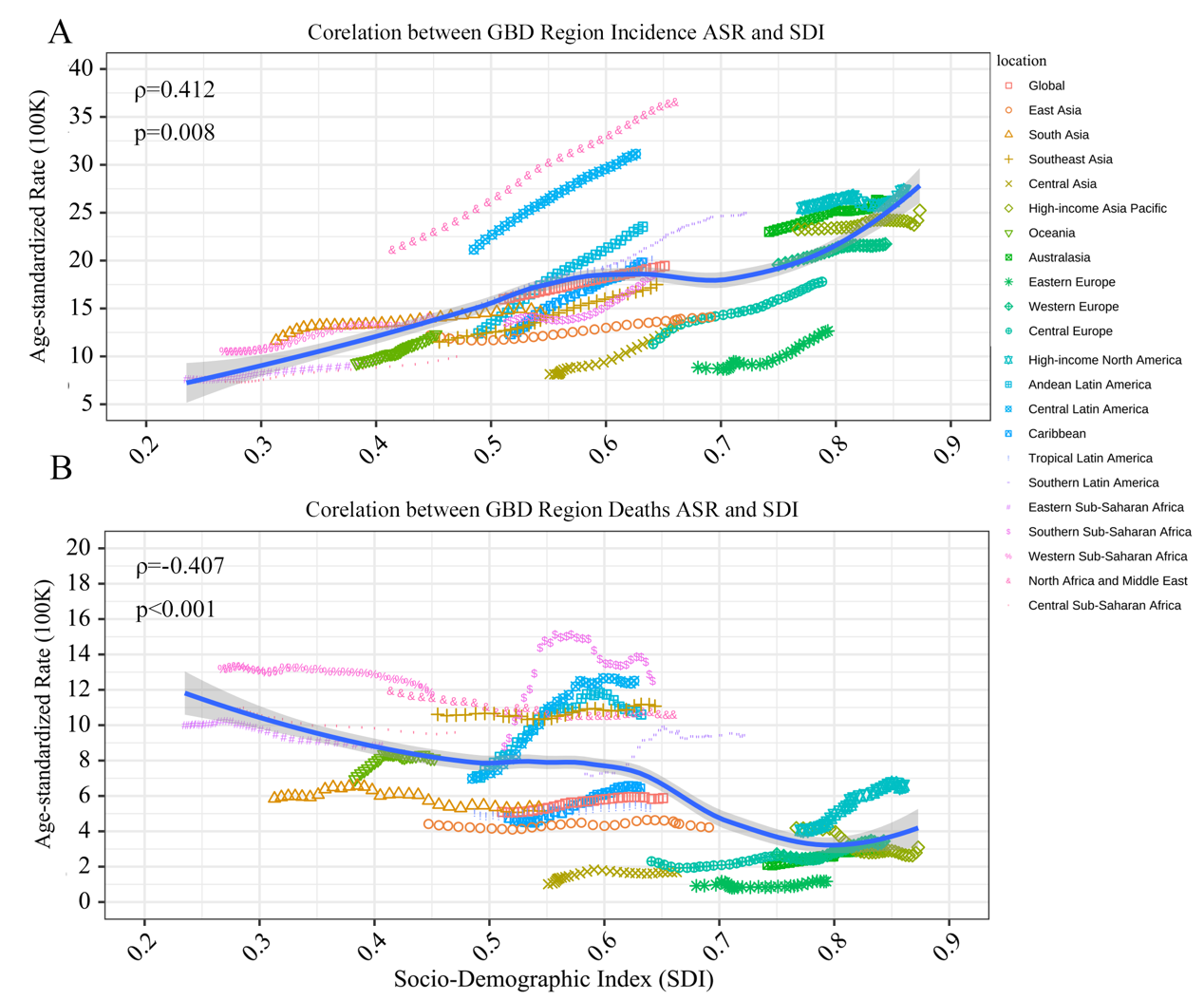


**Supplementary Figure 5.** The socioeconomic disparities of age-standardized incidence (A) and deaths (B) rates of CKDs due to hypertension in 21 GBD geographic regions.

**
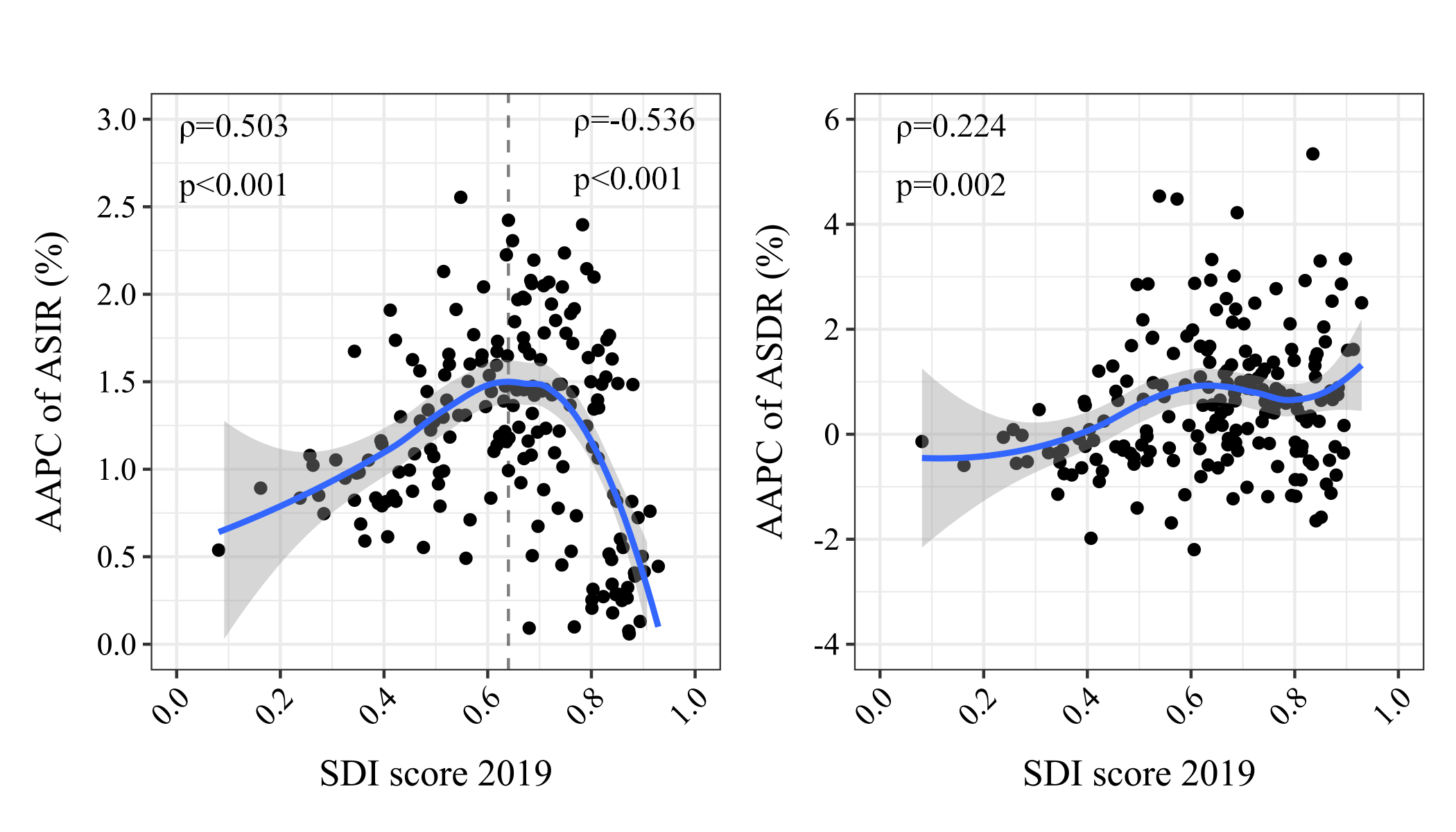
**

**Supplementary Figure 6.** The correlation between social-demographic index (SDI) and average annual percentage changes (AAPC) of age-standardized incidence and deaths rates of CKDs due to hypertension from 1990 to 2019.


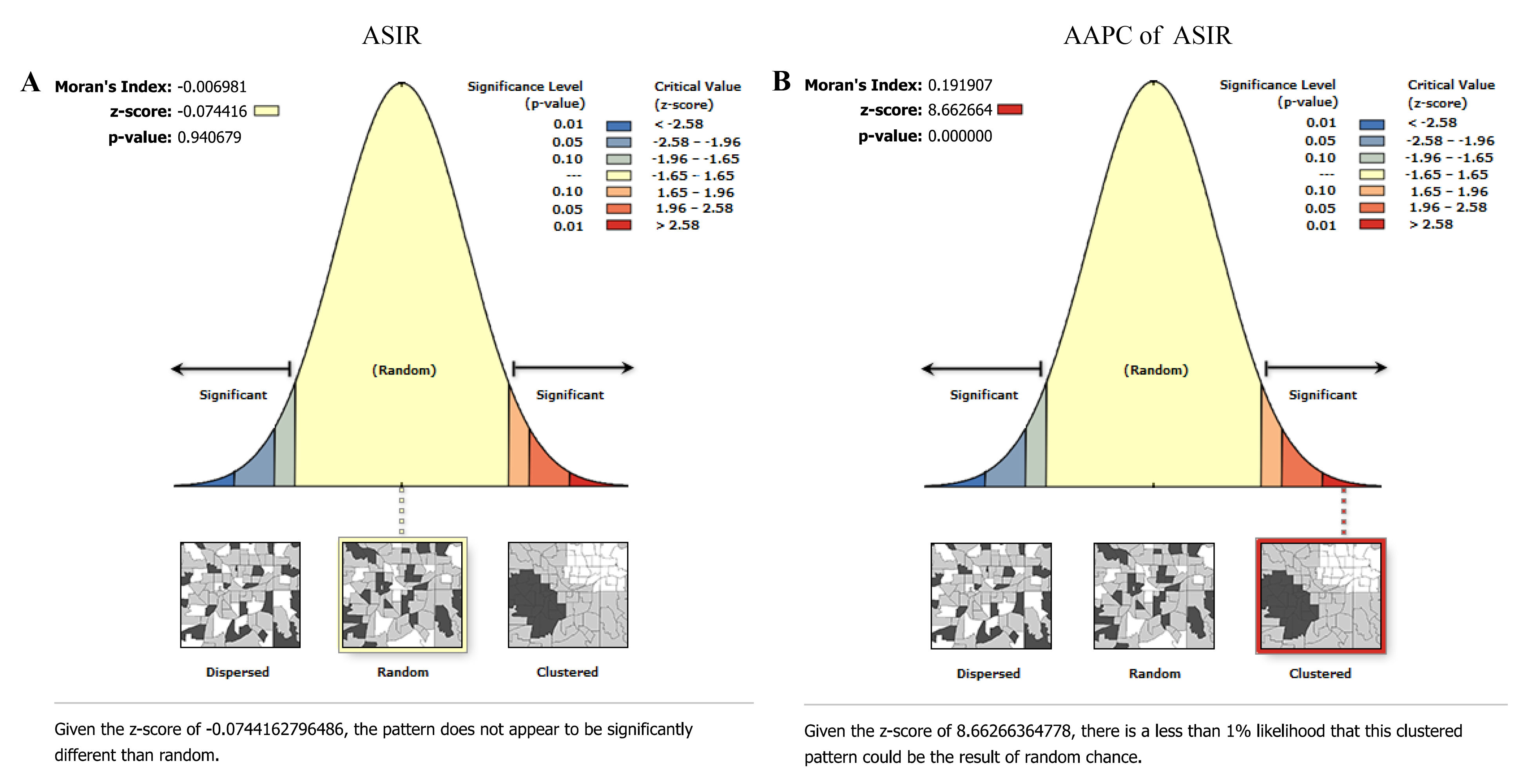


**Supplementary Figure 7.** Final statistics and Moran′s diagram for 204 countries of age standardized incidence rate (ASIR, A) in 2019, and its average annual percentage changes (AAPCs, B) from 1990 to 2019.


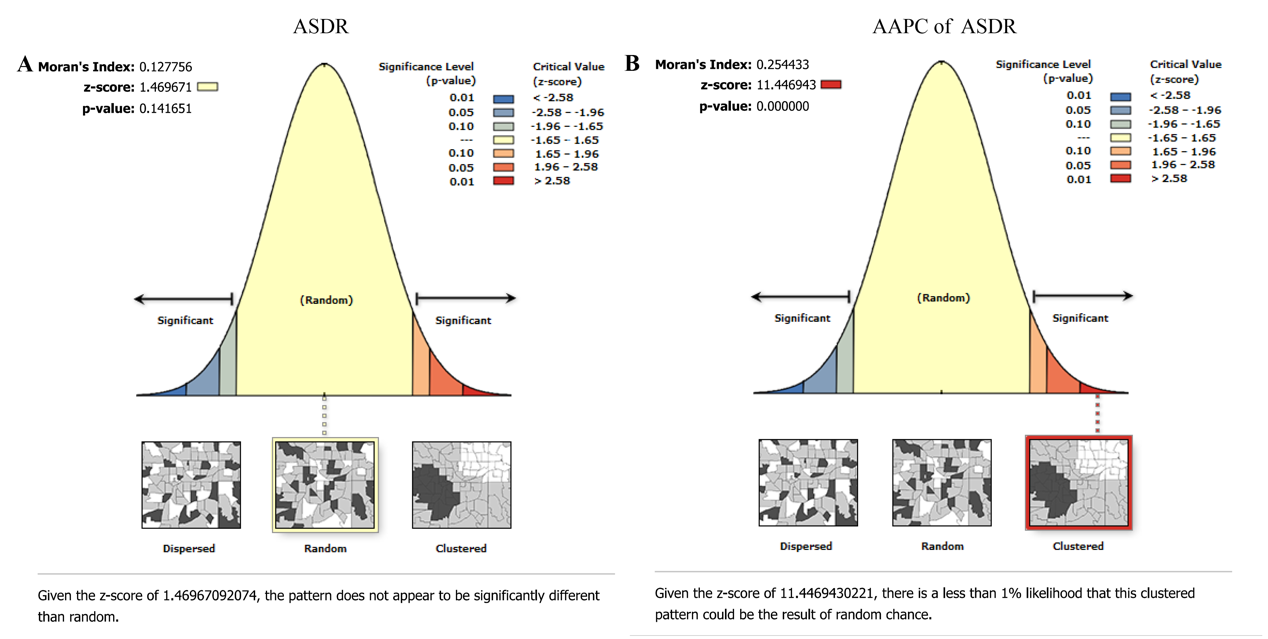


**Supplementary Figure 8.** Final statistics and Moran′s diagram for 204 countries of age standardized death rate (ASDR, A) in 2019, and its average annual percentage changes (AAPCs, B) from 1990 to 2019.
